# Comparative Respiratory Protective Effects of *Auricularia auricula* Polysaccharide Fractions: From Antioxidant-Dependent Antitussive Effects to Microbiota-Associated Anti-Asthmatic Activity

**DOI:** 10.3390/foods15213833

**Published:** 2026-10-27

**Authors:** Sinan Chen, Jun Wang, Fan Li, Lu Wang, Sijia Liu, Xu Zhang, Yi Wan

**Affiliations:** 1 Microbiology Institute of Shaanxi, Xiying Road 76, Xi’an 710043, China; chensinan111@163.com (S.C.);; 2 Shaanxi Provincial Engineering Research Center for Microbial Resources Exploitation and Utilization in Qinba Mountain Area, Xi’an 710043, China

**Keywords:** *Auricularia auricula* polysaccharides, different molecular weights, respiratory protection, antioxidant, NF-κB pathway, gut microbiota

## Abstract

As an edible and medicinal fungus, *Auricularia auricula* contains polysaccharides (AAPs) as its primary bioactive components. The molecular weight (*M*w) of these macromolecules is recognized as a critical determinant of their bioavailability and physiological effects. However, systematic comparative studies on the respiratory protective effects of AAPs with *M*ws are still limited. In this study, two AAPs with distinct *M*ws (1554 kDa and 31 kDa) were prepared and characterized, and their antitussive, expectorant, and anti-asthmatic activities were evaluated. The results revealed that the two AAPs exhibited different monosaccharide compositions. Compared with AAPs-H (1554 kDa), AAPs-L (31 kDa) showed superior in vitro antioxidant activity, better solubility, and lower viscosity. Furthermore, AAPs-L improved redox profiles (SOD, GSH-Px, MDA) and reduced substance P release to 2.17 pg/mL, thereby attenuating the cascade of pro-inflammatory mediators. Consequently, mice treated with 200 mg/kg BW AAPs-L exhibited a significant reduction in cough frequency and prolonged cough incubation period. In contrast, AAPs-H, with its higher *M*w and mannose content (50.24%), exerted unique anti-asthmatic effects by restoring Th1/Th2 immune balance, modulating the NF-κB signaling pathway, and reshaping the gut microbiota, as evidenced by an increased abundance of *Cryptobacteroides sp900546395*. And both AAPs promoted phenol red excretion, indicating expectorant effects. This study comprehensively evaluated and compared the differential protective effects of AAPs with distinct *M*ws on the respiratory system in mice, providing a new perspective for the functional food application and comprehensive utilization of *M*w-tailored AAPs.

## 1. Introduction

*Auricularia auricula* (L.) Underw. (phylum Basidiomycota, class Agaricomycetes, order Auriculariales, family Auriculariaceae; Index Fungorum) is a well-documented edible and medicinal fungus, valued for its rich nutritional profile and therapeutic potential [1]. It contains various bioactive components, including polysaccharides, dietary fiber, and proteins. Among these, *A. auricula* polysaccharides (AAPs) represent the primary active constituents [2,3], exhibiting a spectrum of bioactivities such as immunomodulation [4], antitumor [5], anti-inflammatory [6], antioxidant [7], and lipid-lowering [8] properties. Importantly, polysaccharides constitute a broad class of macromolecules whose molecular weight (*M*_W_) is considered a decisive factor influencing their bioavailability, receptor interactions, and subsequent physiological effects [9]. Meanwhile, the high degree of polymerization, structural complexity, elevated viscosity, and limited solubility of high-*M*_W_ polysaccharides often restrict their processing and application. Consequently, preparation strategies and structural characteristics of AAPs with different *M*_W_ ranges have been extensively investigated [10,11], although these polysaccharides may undergo chemical degradation or structural modification. It has also been suggested that *M*_W_-dependent conformational changes may affect the binding of AAPs to cellular receptors, thereby modulating their bioactivity [12]. Furthermore, monosaccharide composition is likely another critical factor in the structure-activity relationship of polysaccharides. For instance, Li et al. prepared a glucuronomannan containing 91% mannose (Man) [13], which promoted B-lymphocyte proliferation and enhanced nitric oxide production in macrophages. Nevertheless, to our knowledge, no systematic comparative study has yet reported how *M*_W_ influences the protective efficacy of AAPs against respiratory diseases.

Respiratory diseases are common clinical disorders characterized by symptoms such as cough, sputum production, wheezing, and pulmonary imaging abnormalities. Their pathological mechanisms typically involve oxidative stress, chronic inflammation, and immune dysregulation. Excessive generation of reactive oxygen species (ROS) in the airways induces oxidative stress, triggering an inflammatory cascade that involves sustained infiltration and release of various immune cells (e.g., eosinophils, neutrophils) and cytokines (e.g., IL-4, IL-5, IL-13, TNF-α). This process further disrupts the Th1/Th2 immune balance, perpetuating a vicious cycle of inflammation and contributing to conditions such as asthma [14]. Among these, the nuclear factor-κB (NF-κB) pathway occupies a central regulatory position. Accumulating evidence indicated that modulation of the NF-κB signaling pathway could alleviate respiratory inflammation, including asthma [15,16], which had established NF-κB as a key therapeutic target for various herbal medicines [17,18] and plant extracts [19]. Concurrently, emerging studies highlight the critical role of the gut-lung axis in regulating pulmonary immunity and inflammation. For example, Luo et al. recently demonstrated that Morusin ameliorated respiratory infection in a cough rat model by reshaping the gut microbiota and modulating ERK signaling along the gut-lung axis [20]. AAPs have also been shown to attenuate acute lung injury by regulating gut microbiota composition and related metabolic pathways [21]. However, research on whether and how AAPs of different *M*_W_s mediate respiratory protection via the gut-lung axis remains limited.

In this study, two AAP components with different *M*_W_s (1554 kDa and 31 kDa) were prepared and characterized. We hypothesized that AAPs with different *M*_W_s might act through distinct respiratory protective mechanisms: the lower-*M*_W_ fraction (AAPs-L), with its stronger radical-scavenging capacity, would tend to exert antioxidant-dependent antitussive effects, whereas the higher-*M*_W_ fraction (AAPs-H), with its immunomodulatory potential, would tend to alleviate asthma-related airway inflammation via restoring Th1/Th2 balance, modulating the NF-κB pathway, and reshaping the gut microbiota. To test this hypothesis, the antitussive, expectorant, and anti-asthmatic activities of both fractions were evaluated, together with their antioxidant effects, immunomodulation, NF-κB-mediated anti-inflammatory responses, and gut microbiota regulation. This work aims to provide a new perspective for the dietary application and development of *M*_W_-tailored AAPs.

## 2. Materials and Methods

### 2.1. Preparation and Characterization of AAPs

#### 2.1.1. Extraction of AAPs-H and AAPs-L

The fruit bodies of *A. auricula* were ground into powder (passing through an 80-mesh sieve). The powder was mixed with distilled water at a solid-to-liquid ratio of 1:50 (*w*/*v*), stirred thoroughly, and soaked for 30 min. Subsequently, 1.2% (*w*/*w*, based on dry material) cellulase, 0.8% pectinase, and 0.5% neutral protease were added to the mixture. Enzymatic hydrolysis was conducted at 50 °C in a water bath for 1 h. Following enzymatic treatment, hydrogen peroxide (H_2_O_2_) was added to the slurry at a final concentration of 0.07% (*v*/*v*). The mixture was then heated to 100 °C and extracted with continuous stirring for 2 h. The extract was centrifuged at 7000 rpm for 20 min to collect the supernatant. The polysaccharides in the supernatant were precipitated by adding four volumes of absolute ethanol. The resulting precipitate was collected, re-dissolved in distilled water, filtered, and finally spray-dried to obtain the low *M*_W_ AAPs fraction, designated as AAPs-L, whose preparation incorporated oxidative treatment. For comparison, a high *M*_W_ polysaccharide fraction was prepared following the same procedure but without the addition of H_2_O_2_. This fraction was designated as AAPs-H. Store at 4 °C for subsequent analysis and animal experiments. The yields of AAPs-L and AAPs-H were calculated using the following equation:

Yield (%) = (m/M) × 100(1)

where m is the mass (g) of AAPs-L or AAPs-H powder obtained after spray drying, and M is the mass (g) of the initial crude AAPs powder.

#### 2.1.2. Molecular Weight Determination

The *M*_W_s of AAPs-H and AAPs-L were determined by gel permeation chromatography (GPC) using a high-performance liquid chromatography system (HPLC) (Waters, Milford, MA, USA) equipped with a refractive index detector (RID) (model 2414, Waters). Separation was performed on a Waters Ultrahydrogel Linear column (7.8 × 300 mm) maintained at 30 °C. The mobile phase was 0.1 M sodium nitrate solution at a flow rate of 0.5 mL/min. The AAPs samples were prepared at a concentration of 5 mg/mL, filtered through a 0.45 μm membrane, and injected at a volume of 10 μL. A calibration curve was constructed using *n* = 6 dextran standards with known *M*_W_s ranging from 4.3 to 1500 kDa (4.3, 41.4, 72.7, 147.6, 312, and 1500 kDa). The weight-average molecular weight (*M*_W_) of AAPs-H and AAPs-L was calculated based on their retention times using the calibration curve.

#### 2.1.3. Monosaccharide Composition Analysis

The monosaccharide composition of AAPs-L and AAPs-H was analyzed using HPLC coupled with an electrochemical detector (ED). Briefly, AAPs samples were hydrolyzed with 1 mL of 2 M trifluoroacetic acid (TFA) at 121 °C for 2 h. The hydrolyzate was dried under nitrogen stream, washed with methanol, and redissolved in ultrapure water before analysis. A mixed standard solution containing 13 monosaccharides (fucose (Fuc), rhamnose (Rha), arabinose (Ara), galactose (Gal), glucose (Glc), xylose (Xyl), Man, fructose (Fru), ribose (Rib), galacturonic acid (Gal-UA), glucuronic acid (Gul-UA), guluronic acid (Glc-UA), and mannuronic acid (Man-UA)) at various concentrations was prepared for calibration.

Chromatographic separation was performed on a Dionex Carbopac™ PA20 column (3 × 150 mm, 10 μm) maintained at 30 °C. The mobile phases consisted of (A) ultrapure water, (B) 0.1 M NaOH, and (C) 0.1 M NaOH containing 0.2 M NaAc. The flow rate was 0.5 mL/min, and the injection volume was 5 μL. The elution gradient was programmed as follows: 0 min, A/B/C (95:5:0, *v*/*v*); 26 min, A/B/C (85:5:10, *v*/*v*); 42 min, A/B/C (85:5:10, *v*/*v*); 42.1 min, A/B/C (60:0:40, *v*/*v*); 52 min, A/B/C (60:40:0, *v*/*v*); 52.1 min, A/B/C (95:5:0, *v*/*v*); and 60 min, A/B/C (95:5:0, *v*/*v*).

#### 2.1.4. FT-IR Spectroscopy

Infrared spectra of the AAPs-H and AAPs-L were recorded with a Nicolet 170SX FT-IR (Spectrum One, PerkinElmer Co., Shelton, CT, USA) spectrometer in the range 4000 to 400 cm^−1^ using the KBr disk method [22]. Briefly, each lyophilized AAPs-L and AAPs-H sample was ground with dried KBr powder at a mass ratio of 1:100, pressed into a transparent pellet under 40 MPa for 2 min, and scanned in the range of 4000–500 cm^−1^. A pure KBr pellet was used as the background. Measurements were performed in triplicate

#### 2.1.5. Congo Red Test

The NaOH concentration affects the maximum absorption wavelength of the complex formed by Congo red (C_32_H_22_N_6_O_5_S_2_Na_3_) and polysaccharides with a triple helix structure. With slight modifications to the method of Zhang et al. [23], 0.5 mg/mL AAPs-H or AAPs-L sample solutions (1.0 mL) were mixed with 1.0 mL of Congo red solution (50 μmol/L). The pH of the mixture was adjusted by adding NaOH solution at final concentrations of 0, 0.1, 0.2, 0.3, 0.4, and 0.5 mol/L. After equilibration at room temperature for 10 min, the absorbance was scanned from 400 to 600 nm using a microplate reader (BioTek Synergy HTX, Winooski, VT, USA), and λmax was recorded. The mixture of deionized water and Congo red solution was used as the control. All measurements were performed in triplicate.

#### 2.1.6. Scanning Electron Microscope (SEM)

The surface morphology of AAPs-H and AAPs-L was examined by scanning electron microscopy (SEM). Briefly, lyophilized polysaccharide powders were fixed onto conductive carbon tape mounted on aluminum stubs. The samples were then sputter-coated with gold at 20 mA for 60 s. No chemical treatment was applied to the samples before coating. The coated samples were examined with a scanning electron microscope (JSM-7500, JEOL, Tokyo, Japan) at an accelerating voltage of 5 kV and a working distance of 8 mm. Images were captured at 5000× magnification.

### 2.2. Antioxidant Activity

#### 2.2.1. Scavenging Activity of ABTS^+^ Radical

The ABTS^+^ radical scavenging activity of AAPs-L and AAPs-H was assessed following the method described by Re et al. with minor modifications [24]. Briefly, ABTS solution (7.68 mg/mL) was mixed with an equal volume of potassium persulfate solution (1.34 mg/mL) and allowed to react overnight in the dark. Prior to analysis, the mixture was diluted with distilled water to achieve an absorbance of 0.800 ± 0.020 at 734 nm. A 0.4 mL aliquot of sample solution was mixed with 0.5 mL of ABTS working solution and incubated in the dark for 7 min. The absorbance (A_1_) was measured at 734 nm using a microplate reader (BioTek Synergy HTX, Winooski, VT, USA). A_2_ was determined by replacing the ABTS solution with distilled water, and A_0_ was determined by replacing the sample solution with distilled water. Vitamin C (Vc) was used as a positive control. The scavenging rate was calculated using the following equation:

ABTS^+^ radical scavenging % = [1 − (A1 − A2)/A0] × 100% (2)

#### 2.2.2. Scavenging Activity of DPPH Radical

The DPPH radical scavenging activity was evaluated based on the method of Shimada et al. with slight modifications [25]. A DPPH ethanol solution (4.3 mM) was prepared and diluted to obtain an absorbance of 0.800 ± 0.020 at 517 nm. A 0.5 mL aliquot of sample solution was mixed with 0.5 mL of DPPH solution and incubated in the dark for 20 min. The absorbance (A_1_) was measured at 517 nm using a microplate reader (BioTek Synergy HTX, Winooski, VT, USA). A_2_ was determined by replacing the DPPH solution with anhydrous ethanol, and A_0_ was determined by replacing the sample solution with distilled water. Vc served as a positive control. The scavenging rate was calculated as follows:

DPPH radical scavenging % = [1 − (A1 − A2)/A0] × 100%(3)

#### 2.2.3. Scavenging Activity of Superoxide Anion (O_2_^−^•)

The method of Markulund modified by Ekanayake et al. was used in this test [26,27]. The method is based on the inhibition of the autoxidation of pyrogallol by antioxidant components. Add 0.4 mL of sample solution and a freshly prepared solution of pyrogallol (0.1 mL) to Tris-HCl buffer (0.6 mL). The autoxidation reaction rate of pyrogallol was determined at 325 nm by monitoring the absorbance every 30 s for a total period of 10 min, corresponding to the end of the reaction. Measure the absorbance values A0 and A1 of the sample at 325 nm, and simultaneously measure the absorbance values A2 and A3 at 325 nm using water as a substitute for the sample. Using Vc as the control, and the scavenging activity of O_2_^−^• was calculated by the following formula:

O_2_^−^ radical scavenging % = [1 − (A1 − A0)/(A3 − A2)] × 100% (4)

#### 2.2.4. Determination of Reducing Power

The reducing power of the test samples was determined by the method of Yen and Duh [28]. Mix 0.1 mL each of sample solutions at different concentrations (1, 3, 5, 7, 9 mg/mL), 1% potassium ferricyanide, and 0.2 mol/L phosphate buffer (pH 6.6) uniformly. The mixtures were incubated for 20 min at 50 °C. After cooling in an ice bath for 30 s, add 0.1 mL of concentrated hydrochloric acid. Centrifuge (4 °C, 4000 rpm, 20 min). The upper layer (0.15 mL) was mixed with 0.5 mL of distilled water and 0.05 mL of 0.1% ferric chloride and the absorbance was measured at 700 nm. The positive control is ascorbic acid.

### 2.3. Experimental Animals and Treatments

BALB/C mice (20 ± 2 g) were purchased from ZhuHai Bestest Biotechnology Co., Ltd. (Zhuhai, Guangdong, China) (SYXK (Guangdong) 2023-002). All mice were housed under standard specific pathogen-free (SPF) conditions with a controlled temperature (22 ± 2 °C), relative humidity (50 ± 10%), and a 12/12 h light/dark cycle. All animal experiments were strictly performed in accordance with the National Institutes of Health Guide for the Care and Use of Laboratory Animals, and were approved by the Institutional Ethical Committee for Animal Research of Institute of Zoology, Guangdong Academy of Sciences. (Approval No.: GIZ20250314-03).

The anti-tussive, expectorant, and anti-asthmatic experiments were performed as three independent experiments, and separate cohorts of mice were used for each experiment. After a 7-day acclimatization period, mice in each experiment were randomly allocated into four groups (*n* = 8 per group) for oral gavage administration as follows: Model group received no treatment. Positive control group administered standard reference drugs, including pentoxyverine citrate (P: 10 mg/kg body weight (BW)) for antitussive assays, NH_4_CL (1 g/kg BW) for expectorant evaluation, or dexamethasone (DEX: 3 mg/kg BW) for anti-asthmatic experiments, depending on the specific experimental model. Low-molecular-weight AAPs group (AAPs-L) received AAPs-L at a dose of 200 mg/kg BW. High-*M*w AAPs group (AAPs-H) received AAPs-H at a dose of 200 mg/kg BW. In all experiments, changes in mice body weight were recorded daily. 

### 2.4. Anti-Tussive Effect Assessment

The antitussive activity was evaluated using a classical ammonia liquor-induced cough model in mice. Following the acclimatization period, all male BALB/C mice were initially subjected to cough induction by exposure to nebulized 25% ammonia liquor in an ultrasonic nebulizer, and the number of coughs within 3 min was recorded. Mice exhibiting fewer than 20 coughs were excluded. The eligible mice were then randomly assigned to the four groups above. Each group received oral gavage once daily for 7 consecutive days, while the model group was administered an equal volume of physiological saline. Twenty-four hours before the final administration, all mice were fasted with free access to water. One hour after the last treatment, all mice were individually placed in the nebulizer chamber and exposed to nebulized 25% ammonia liquor to induce coughing. The latency to the first cough and the cough number of coughs within 3 min were recorded. Following the cough assessment, the mice were euthanized and subjected to tracheal cannulation. Bronchoalveolar lavage fluid (BALF) was collected by instilling 0.6 mL of physiological saline into the lungs twice via the tracheal cannula. The recovered lavage fluids were pooled and centrifuged at 1000 r/min for 5 min at 4 °C. The supernatant was aliquoted and stored at −80 °C for further analysis. After BALF collection, systemic perfusion via the heart was performed to clear blood from the organs. Subsequently, the lungs, thymus, and liver were excised. Lung tissues were snap-frozen on dry ice and stored at −80 °C. The thymus and liver were weighed to calculate the relative mass of liver and thymus (organ weight/body weight × 100%).

### 2.5. Expectorant Effect Assessment

Male BALB/C mice were randomly allocated into the four groups described above. The experiment was conducted according to the previously described method with minor modifications [29]. Briefly, each group received oral gavage once daily for 7 consecutive days. One hour after the final administration, mice were intraperitoneally injected with 5% phenol red solution at a volume of 0.2 mL/10 g body weight. After 30 min, mice were euthanized and subjected to tracheal cannulation. The trachea was lavaged twice with 0.6 mL of 5% NaHCO_3_ solution each time, and the lavage fluid was collected and pooled. The BALF was centrifuged at 1000 r/min for 5 min at 4 °C. The supernatant was collected, and its absorbance was measured at 558 nm on an enzyme labeler (BioTek Synergy HTX, Winooski, VT, USA). The phenol red concentration was then calculated based on a standard curve.

### 2.6. The Anti-Asthmatic Effect Assessment

Female BALB/c mice showing appropriate sensitivity in preliminary screening (based on established criteria from previous studies) were selected (*n* = 5) for the formal experiment [29]. The selected mice (6–8 weeks old) were randomly allocated into the four groups described above, and were sensitized by intraperitoneal injection of 25 µg OVA (Grade V, Sigma-Aldrich, St. Louis, MO, USA) adsorbed on 1 mg aluminum hydroxide gel in 200 µL PBS on days 0 and 7. From days 14 to 20, mice were exposed to aerosolized 1% (*w*/*v*) OVA for 30 min daily using an ultrasonic nebulizer. AAPs-L, AAPs-H or DEX were administered daily by oral gavage from day 0 until the end of the experiment (day 21). The model group received an equivalent volume of PBS. On day 21, 24 h after the final OVA challenge, mice were exposed to nebulized aerosol generated from an equal-volume mixture of 0.1% histamine phosphate and 2% acetylcholine to assess airway hyperresponsiveness. The latency to asthma-like symptoms was recorded. After 24 h, all mice were euthanized via intraperitoneal injection of an overdose of 3% sodium pentobarbital. Blood was collected via cardiac puncture, followed by tracheal cannulation. The lungs were lavaged twice with 0.6 mL of physiological saline each time, and the recovered BALF was pooled. The pooled BALF was centrifuged at 1000 r/min for 5 min at 4 °C, and the supernatant was collected and stored at −80 °C for further analysis. After BALF collection, systemic cardiac perfusion with physiological saline was performed. The lungs and spleen were then excised. Lung tissues were immediately snap-frozen on dry ice and stored at −80 °C. The spleen was weighed, and the relative mass of spleen was calculated.

### 2.7. Biochemical Analysis 

To evaluate the anti-inflammatory, antioxidant, and immunomodulatory activities of AAPs, biochemical analyses were performed. In the anti-tussive assessment, concentrations of substance P (SP), chemokine (C-X-C motif) ligand 1 (CXCL1), and interleukin-1β (IL-1β) in BALF were measured using commercially available ELISA kits (Byabscience Biotechnology Co., Ltd., Nanjing, China). The activities of superoxide dismutase (SOD) and glutathione peroxidase (GSH-Px), as well as the levels of malondialdehyde (MDA) and nitric oxide (NO) in lung tissue, were determined with corresponding assay kits (Byabscience Biotechnology Co., Ltd., Nanjing, China). In the anti-asthmatic assessment, levels of tumor necrosis factor-alpha (TNF-α), IL-1β, and IL-4 in BALF, together with serum immunoglobulin E (IgE) levels, were quantified by ELISA purchased from Byabscience Biotechnology Co., Ltd. (Nanjing, China). All procedures were conducted in strict accordance with the manufacturers’ protocols.

### 2.8. Histopathological Analysis

After the animals were sacrificed, lung tissues were collected from mice in each group. The entire lung was fixed in 4% paraformaldehyde solution at room temperature for 24 h, dehydrated through a graded ethanol series, cleared in xylene, and embedded in paraffin. Paraffin-embedded blocks were sectioned at a thickness of 4 μm. After deparaffinization and rehydration, the sections were stained with hematoxylin for 5 min and eosin for 5 s. Finally, the sections were examined with a microscope (Olympus CX22LED, Hachioji, Tokyo, Japan) at 100× and 400× [30], and to assess the effects of different molecular-weight AAPs on histopathological changes in asthmatic mice.

### 2.9. Quantification of Gene Expression Analysis

Using the Takara MiniBEST Universal RNA Extraction Kit (Takara, Dalian, China), total RNA was isolated from lung tissues, and the purity of the RNA was detected by spectrophotometry (Thermo NanoDrop 2000, Shanghai, China) determining 260/280 nm absorbance ratio. Complementary DNA (cDNA) was synthesized from the extracted RNA using the Perimscript^TM^ rt master mix kit (Takara, Dalian, China). Quantitative real-time PCR (qPCR) was subsequently performed with TB Green™ Premix Ex Taq™ II (Takara, Dalian, China) on a qPCR amplification instrument (Bio-rad CFX Connect, Hercules, CA, USA). The protocol consisted of an initial denaturation at 95 °C for 30 s, followed by 40 cycles of denaturation at 95 °C for 5 s and annealing/extension at 60 °C for 30 s [31]. Primer sequences used for amplifying TNF-α, NF-κB p65, IL-1β, and the reference gene β-actin were as follows: TNF-α: Forward 5′-ACGTGGAACTGGCAGAAGAG-3′, Reverse 5′-CTCCTCCACTTGGTGGTTTG-3′; IL-1β: Forward 5′-CTCCATGAGCTTTGTACAAGG-3′, Reverse 5′-TGCTGATGTACCAGTTGGGG-3′; NF-κB p65: Forward 5′-ATGGCAGACGATGATCCCTAC-3′, Reverse 5′-CGGAATCGAAATCCCCTCTGTT-3′; β-actin: Forward 5′-CATGGATGACGATATCGCT-3′, Reverse 5′-CATGAGGTAGTCTGTCAGGT-3′. Gene expression levels were normalized to β-actin and quantified using the 2^−ΔΔCt^ method [32].

### 2.10. Western Blotting Analysis

To evaluate the impact of AAPs on NF-κB signaling transduction, the protein expression levels of NF-κB p65 and its phosphorylated form (p-p65) in mouse lung tissues were analyzed by Western blot. Briefly, approximately 100 mg of lung tissue was homogenized and lysed, followed by centrifugation (4 °C, 12,000 rpm, 20 min). The supernatant was collected, and the protein concentration was determined using a BCA protein assay kit (Nanjing Jiancheng Bioengineering Institute, Nanjing, Jiangsu, China). Equal amounts of protein from each sample were separated by 8% SDS-PAGE and then transferred onto a PVDF membrane (0.55 A, 20 min). After blocking with 5% skim milk overnight, the membrane was incubated with primary antibodies against p-p65 (1:2000), p65 (1:1000), and β-actin (1:2000) for 2 h, followed by extensive washing with TBST. Subsequently, the membrane was incubated with a horseradish peroxidase-conjugated secondary antibody for 1 h and washed again with TBST. The above antibodies were purchased from Proteintech (Wuhan, China) and Wanleibio Technology (Shenyang, China). Protein bands were visualized using enhanced chemiluminescence (EMD Millipore Corporation, Burlington, MA, USA) and imaged with a Gel Image system (Tanon 5200, Shanghai, China). The band intensity of each target protein was quantified relative to β-actin using ImageJ software (version 1.53e, National Institutes of Health, Bethesda, MD, USA).

### 2.11. Gut Microbiota Analysis

Fresh intestinal content samples were collected at the end of the experiment and stored at −80 °C. Total genomic DNA samples were extracted using the MagBeads FastDNA Kit (116564384) (MP Biomedicals, Irvine, CA, USA), following the manufacturer’s instructions, and stored at −20 °C prior to further analysis. The quantity and quality of extracted DNAs were measured using a NanoDrop NC2000 spectrophotometer (Thermo Fisher Scientific, Waltham, MA, USA) and agarose gel electrophoresis, respectively. PCR amplification of the bacterial 16S rRNA genes V3-V4 region was performed using the forward primer 338F (5′-ACTCCTACGGGAGGCAGCA-3′) and the reverse primer 806R (5′GGACTACHVGGGTWTCTAAT-3′). After the individual quantification step (the Quant-iT PicoGreen dsDNA Assay Kit, Invitrogen, Carlsbad, CA, USA), amplicons were pooled in equal amounts, and pair-end 2250 bp sequencing was performed using the Illumina NovaSeq platform with NovaSeq 6000 SP Reagent Kit (500 cycles) at Shanghai Personal Biotechnology Co., Ltd. (Shanghai, China). Sequence data analyses were mainly performed using QIIME2 and R packages (v4.3.3). Raw sequences were processed using QIIME2 and DADA2 for quality filtering, denoising, and amplicon sequence variant (ASV) calling. 

ASV-level alpha diversity indices, such as Shannon diversity index, Simpson index, Pielou’s evenness were visualized as box plots. Beta diversity analysis was performed to investigate the structural variation in microbial communities across samples using Bray–Curtis metrics and visualized via t-Distributed Stochastic Neighbor Embedding (t-SNE). At the genus and species levels, Kruskal–Wallis tests were performed to compare abundance differences among taxonomic units across groups. *p*-values were corrected, and post hoc analyses were conducted to select species with significant abundance differences for visualization. Microbial functions were predicted by PICRUSt2 upon KEGG (https://www.kegg.jp/, accessed on 30 July 2026) databases.

### 2.12. Statistical Analysis

All data are presented as the mean ± SD. Preparations and measurements of AAPs samples were conducted in triplicate. Data statistics and analysis were performed using Microsoft Excel (Microsoft Corp., Redmond, WA, USA) and Origin 2024 (OriginLab Corp., Northampton, MA, USA). The effects of AAPs on biochemical parameters, gene expression, alpha diversity index, etc., were analyzed using the One-way ANOVA with Tukey’s test of GraphPad Prism 6.0 (GraphPad Software, San Diego, CA, USA). And one-way analysis of variance (ANOVA) followed by Tukey’s post hoc test was used for multiple comparisons. For non-normally distributed data, the Kruskal–Wallis test followed by Dunn’s post hoc test was applied. The differences at *p* < 0.05 were considered to be significant.

## 3. Results 

### 3.1. Compositional Analysis and Structural Characterization of AAPs with Different Molecular Weights

The yield of AAPs-H was 14.8%, whereas the yield of AAPs-L obtained after H_2_O_2_ treatment reached 45.7% (Figure S1), representing more than a two-fold increase compared to AAPs-H. This difference may reflect either a naturally higher content of low-molecular-weight polysaccharides in the raw material or, more likely, the degradation of high-molecular-weight polysaccharides into lower-molecular-weight fragments during H_2_O_2_ treatment. 

**Figure 1:**
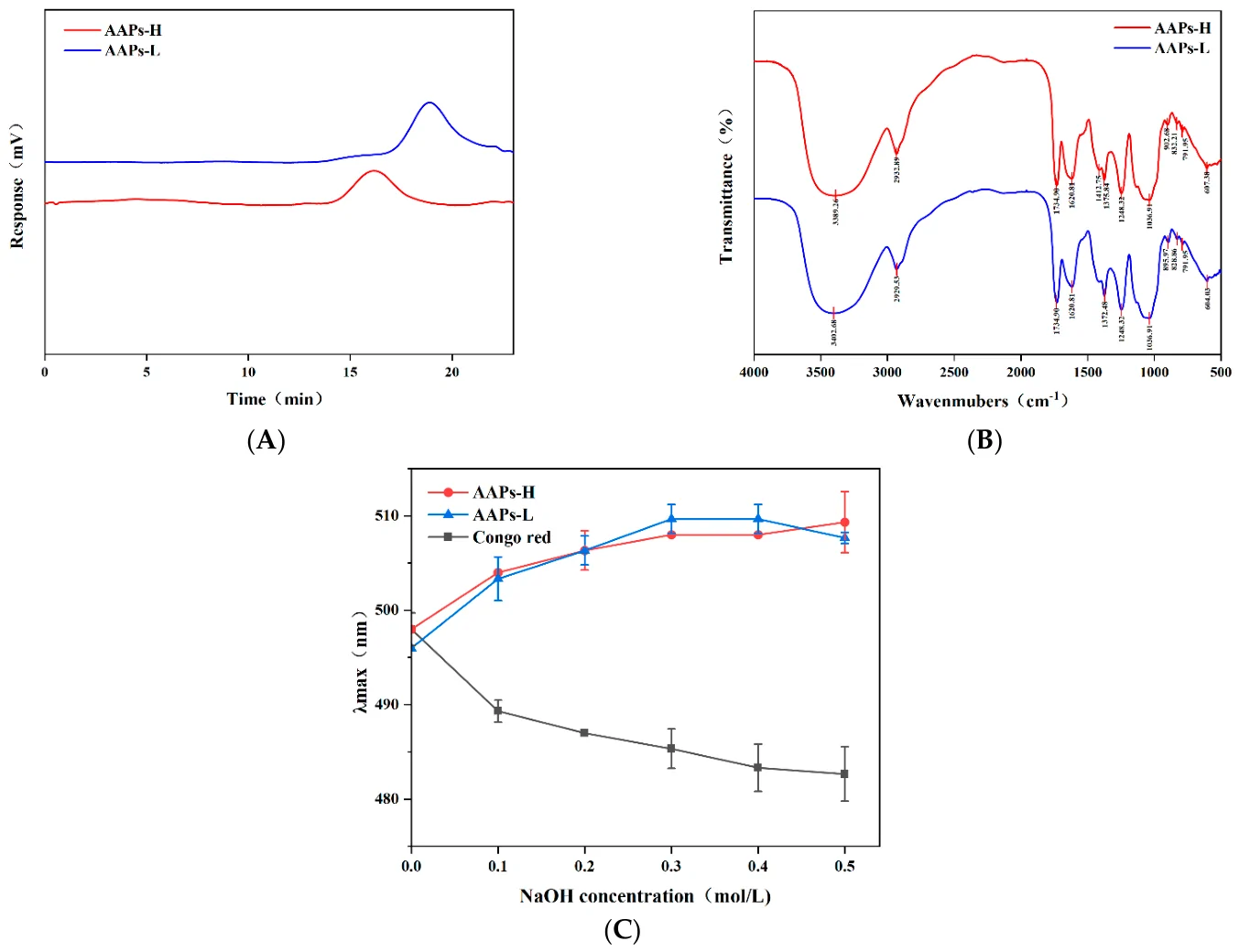

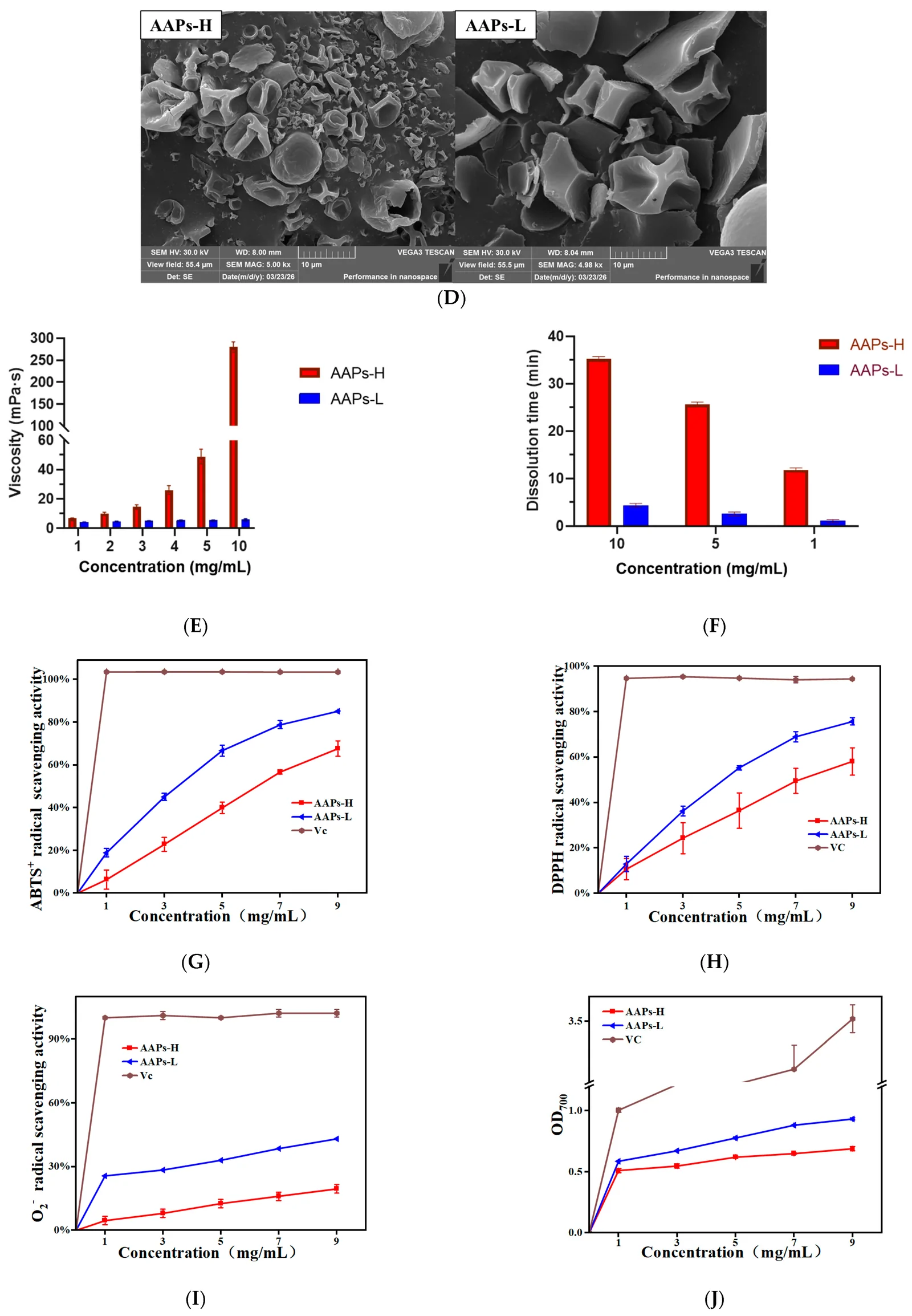
Physicochemical characterization of *Auricularia auricula* polysaccharides (AAPs) with different molecular weights (*M*ws). (**A**) Gel permeation chromatography (GPC) chromatograms, (**B**) Fourier transform infrared (FT-IR) spectra, (**C**) maximum absorption wavelength in NaOH solutions of varying concentrations, (**D**) microscopic structures observed by scanning electron microscopy (SEM) (magnification 5000×), (**E**) viscosity, (**F**) solubility; (**G**) ABTS^+^ radical scavenging activity, (**H**) DPPH radical scavenging activity, (**I**) O_2_^−^ radical scavenging activity, (**J**) reducing power.

The GPC profiles of AAPs-H and AAPs-L were shown in Figure 1A. The retention time of AAPs-L (18.839 min) was shifted to a later time compared to that of AAPs-H (16.139 min), indicating cleavage of the polysaccharide chains upon H_2_O_2_ treatment. Based on the calibration curve, the *M*w of AAPs-H and AAPs-L were calculated to be 1554 kDa and 31 kDa, respectively.

Monosaccharide composition was determined by comparison with a mixture of 13 monosaccharide standards (Figure S2A, Table S1). The molar ratios of individual monosaccharides in AAPs-H (Figure S2B) and AAPs-L (Figure S2C) were calculated from the peak areas in the chromatograms, and were summarized in Table 1. AAPs-H consisted predominantly of Man, Glc, Glc-UA, and Xyl in a molar ratio of 0.50:0.24:0.11:0.10, along with minor amounts of galactose, fucose, arabinose, and rhamnose. AAPs-L was mainly composed of Glc, Man, Xyl, and Glc-UA in a molar ratio of 0.46:0.36:0.08:0.07, with small quantities of galactose, fucose, arabinose, and rhamnose. It can be seen that, compared to AAPs-H, AAPs-L exhibited a higher Glc ratio (46.01%) and a lower Man ratio (36.33%) in its monosaccharide composition, which was significant for its physicochemical properties.

The structural features of AAPs-H and AAPs-L were further elucidated by FT-IR spectroscopy (Figure 1B). Both AAPs-H and H_2_O_2_-treated AAPs-L exhibited typical polysaccharide absorption patterns. A broad and intense absorption band at approximately 3400 cm^−1^ was attributed to O-H stretching vibrations, indicating the presence of extensive intra- and intermolecular hydrogen bonds. A weak band at around 2930 cm^−1^ corresponded to C-H stretching vibrations, confirming the presence of a carbohydrate backbone. The absorption peak at 1370 cm^−1^ was assigned to C-H bending vibrations, which, together with the C-H stretching bands, are characteristic of sugar rings. The absorption at 1735 cm^−1^ was attributed to C=O stretching vibrations, suggesting the presence of uronic acids or ester groups, consistent with an acidic polysaccharide. In the 1200–1000 cm^−1^ region, a strong and broad band at 1037 cm^−1^ was characteristic of C-O-C and C-O-H stretching vibrations in the pyranose ring, serving as the sugar ring. Additionally, an absorption band at 890 cm^−1^ suggested the predominance of β-glycosidic linkages, while a weak band at 830 cm^−1^ indicated the possible presence of minor α-glycosidic linkages or specific Man configurations. Comparison of the FT-IR spectra of AAPs-H and AAPs-L revealed that the positions and shapes of the major characteristic absorption bands were largely consistent, indicating that the polysaccharide backbone structure was preserved after H_2_O_2_-induced degradation. However, a slight decrease in the intensity of the absorption band at 1620 cm^−1^ was observed for AAPs-L, which may be attributed to reduced *M*_W_ and altered spatial conformation, leading to diminished hydrogen-bonded bound water content, potentially affecting solubility and viscosity.

The conformational characteristics of AAPs-H and AAPs-L were further investigated using the Congo red assay (Figure 1C). The control Congo red solution exhibited a concentration-dependent blue shift, with the maximum absorption wavelength (λmax) decreasing from approximately 498 nm at 0 M NaOH to approximately 483 nm at 0.5 M NaOH. In contrast, both AAPs-H and AAPs-L displayed a progressive increase in λmax with increasing NaOH concentration, along with a marked red shift compared to the Congo red control. These results indicated that both AAPs possess a stable triple-helical conformation, and no difference in the spectral shift pattern was observed between AAPs-H and AAPs-L, suggesting that H_2_O_2_ treatment did not disrupt the triple-helical structure of AAPs.

SEM was employed to visualize the micromorphological differences between AAPs-H and AAPs-L (Figure 1D). Micrographs revealed that AAPs-H primarily consisted of small, uniformly dispersed particles, exhibiting a concave surface morphology. But AAPs-L displayed irregular fragments within the concave regions, which were densely packed and aggregated. These observations indicated that the two fractions differ in their microscopic morphology; however, because SEM images reflect the morphology of dried, gold-coated samples, they may not directly reveal the behavior of polysaccharide chains in solution.

### 3.2. Physical Properties and Antioxidant Activity of AAPs with Different Molecular Weights

The viscosity and dissolution properties of AAPs-H and AAPs-L were compared across different concentrations. AAPs-H exhibited a pronounced concentration-dependent increase in viscosity, reaching 280 mPa·s at 10 mg/mL, while AAPs-L maintained a consistently low viscosity (<10 mPa·s) throughout the tested range (Figure 1E). Correspondingly, AAPs-H displayed significantly longer dissolution times at all concentrations (e.g., ~35 min at 10 mg/mL) compared to AAPs-L (<5 min at all tested concentrations) (Figure 1F). These results indicated that compared to high *M*_W_ AAPs, low *M*_W_ AAPs exhibited superior solubility and fluidity. Coupled with its higher yield, this finding holds significant implications for the development and utilization of AAPs-L.

The in vitro antioxidant activity of AAPs with different *M*_W_s was preliminarily evaluated using ABTS^+^ radical scavenging, DPPH radical scavenging, O_2_^−^• scavenging, and reducing power assays. Both AAPs-H and AAPs-L exhibited dose-dependent antioxidant activity across all tested assays, albeit significantly lower than that of Vc. Notably, AAPs-L demonstrated markedly stronger radical scavenging capacity compared to AAPs-H. At a concentration of 9 mg/mL, the ABTS^+^ radical scavenging rate of AAPs-L reached 85.21%, which was 17.51% higher than that of AAPs-H (67.7%) at the same concentration (Figure 1G). Similarly, the DPPH radical scavenging rate of AAPs-L at 9 mg/mL was 75.69%, whereas AAPs-H achieved only 58.15% (Figure 1H). Excessive accumulation of O_2_^−^• can attack cell membranes, proteins, enzymes, and DNA, thereby inducing oxidative stress. At 9 mg/mL, the O_2_^−^ radical scavenging rates of AAPs-H and AAPs-L were 19.54% and 39.12%, respectively (Figure 1I). In the reducing power assay, AAPs-L (0.9337) also exhibited superior activity compared to AAPs-H (0.6897) in 9 mg/mL (Figure 1J), indicating that AAPs-L possessed a greater ability to release hydrogen atoms through cleavage of free radical chain segments.

Therefore, AAPs-L exhibited significantly superior in vitro antioxidant activity compared to AAPs-H in terms of both radical scavenging and reducing capacity. This suggested that *M*_W_ is a critical determinant of the antioxidant activity of AAPs and implied that AAPs-L may more effectively alleviate oxidative stress-related disorders. Combined with its lower viscosity and greater solubility, this makes it a candidate for use as an adjunctive therapeutic agent.

### 3.3. Effects of AAPs on Anti-Tussive Activity

**Figure 2:**
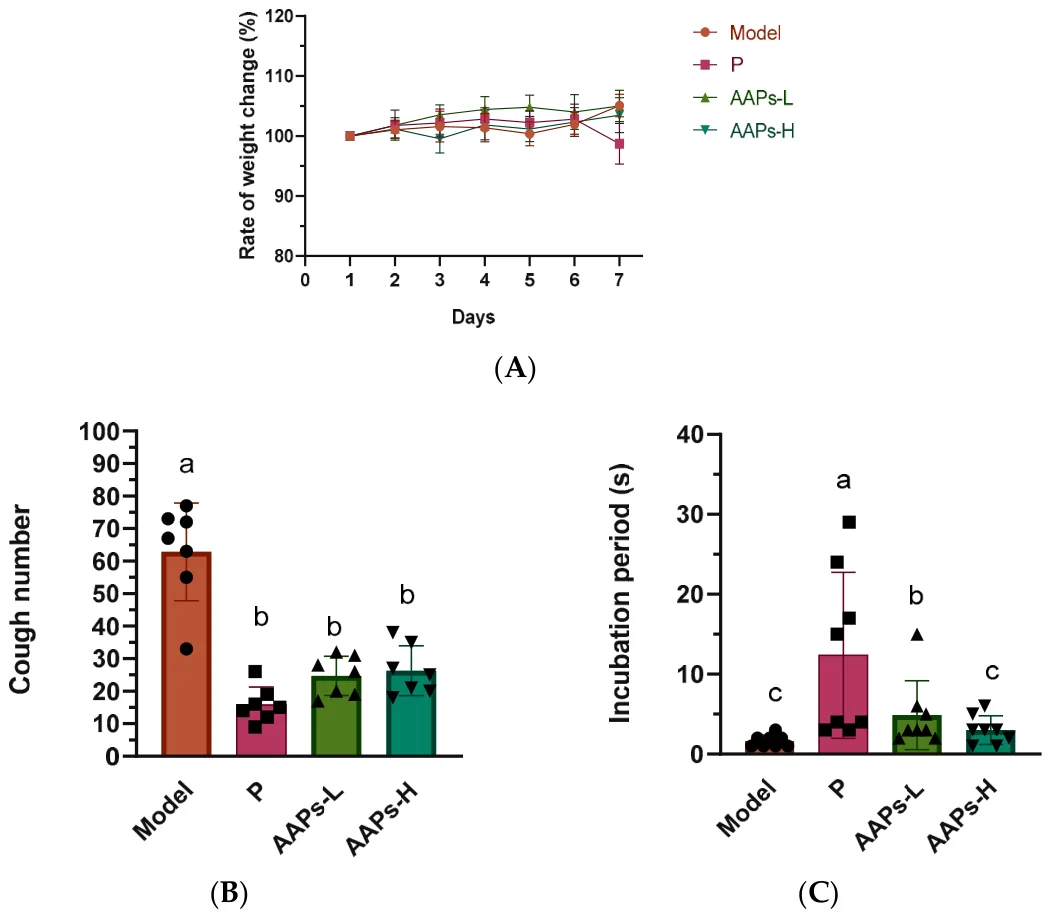
Effect of different molecular weight AAPs on the ammonia liquor induced cough in mice. (**A**) rate of body weight change, (**B**) cough number, (**C**) incubation period. Different lower-case letters correspond to significant differences at *p* < 0.05, *n* = 8.

Body weights remained relatively stable across all groups during both administration and model induction periods, with no significant changes observed. Neither AAPs-L nor AAPs-H administration caused bodily damage leading to weight gain or loss in the mice (Figure 2A). At a dose of 200 mg/kg, the AAPs-L and AAPs-H inhibited coughing by 60.68% and 58.18%, respectively; these results were comparable to the effects of the antitussive agent pentoxyverine citrate (*p* > 0.05) (Figure 2B). The cough incubation period is a time interval that from exposition to ammonia liquor to start coughing. It indicated the potential of the drug on delaying cough [33]. Both the positive and the AAPs-L group showed a significant enhancement in the latent period of cough (*p* < 0.05). Compared with the model group, the latent period of cough in the AAPs-L group increased by 2-fold (Figure 2C), showing a significantly prolonged latency compared with the AAPs-H group. Additionally, due to the potentially short experimental period, no significant differences were observed in the relative mass of liver and thymus among the groups (*p* > 0.05) (Figure S3A,B).

**Figure 3:**
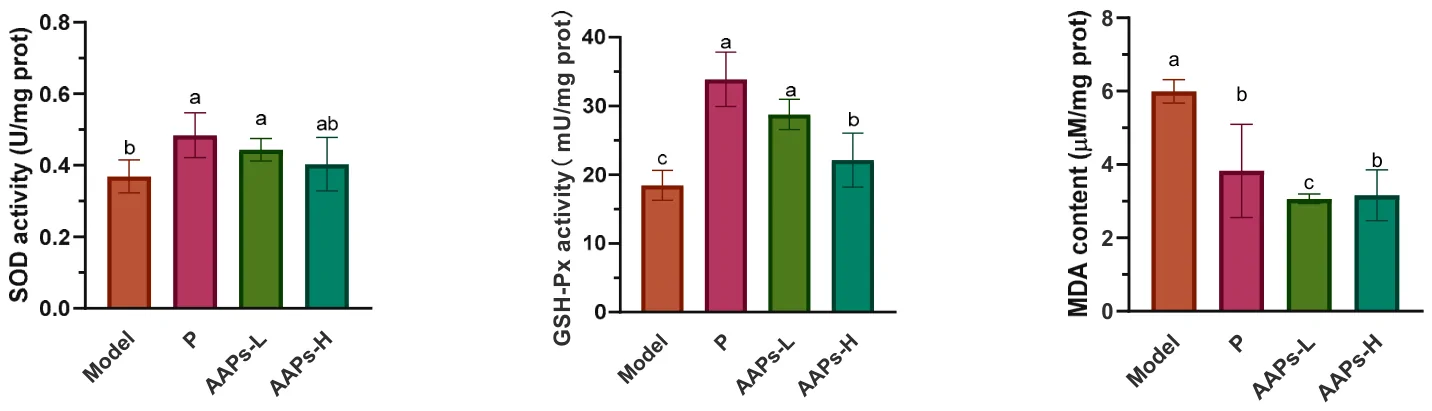

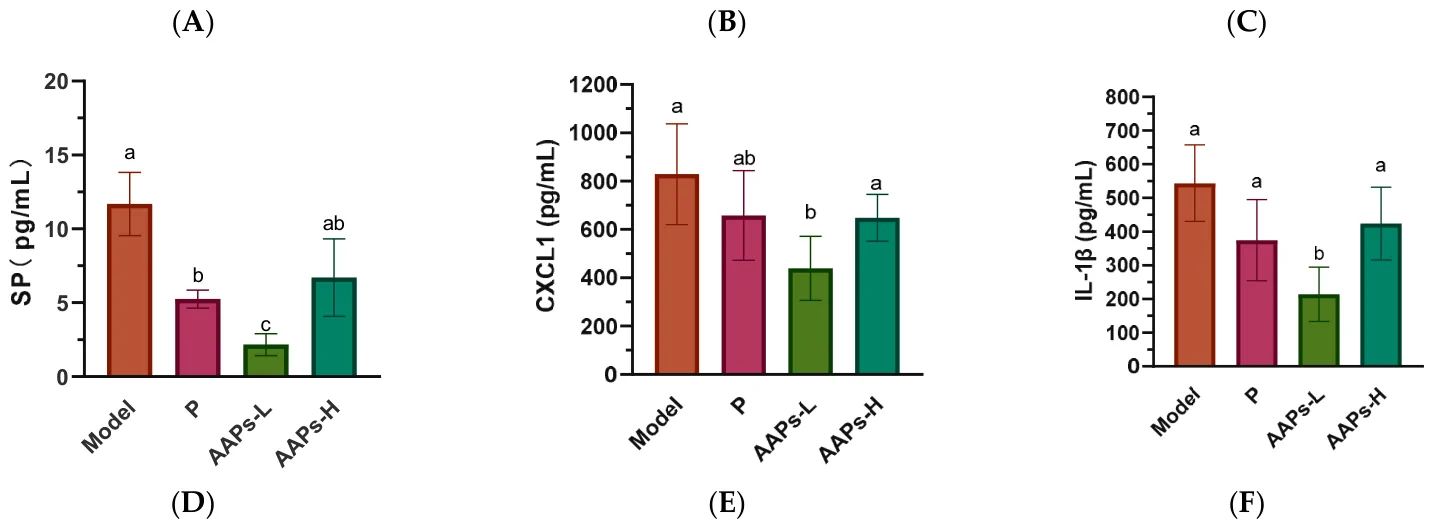
Effects of different molecular weight AAPs on relevant indicators in lung tissue and bronchoalveolar lavage fluid (BALF) on the ammonia liquor induced cough in mice. (**A**) SOD activity, (**B**) GSH-Px activity, and (**C**) MDA content in lung tissue. (**D**) substance P (SP), (**E**) chemokine (C-X-C motif) ligand 1 (CXCL1), and (**F**) interleukin-1β (IL-1β) levels in BALF. Different lower-case letters correspond to significant differences at *p* < 0.05, *n* = 8.

The oxidative stress status in the lungs of coughing mice was evaluated in Figure 3. Ammonia-induced ROS production resulted in significantly lower SOD activity (0.37 U/mg protein) and GSH-Px activity (18.47 mU/mg protein) in the model group compared to other groups, with markedly elevated MDA content (6 μM/mg protein) (*p* < 0.05) (Figure 3C). Both AAPs-L and pentoxyverine citrate reversed this oxidative imbalance. Notably, the MDA content in AAPs-L-treated mice was only 51.11% of that in the cough model group (Figure 3C). Although SOD activity in lung tissue of AAPs-H-treated mice did not increase significantly compared with the model group (Figure 3A) (*p* > 0.05), the GSH-Px activity was significantly elevated (22.13 mU/mg prot) (Figure 3B), and MDA content was significantly reduced (3.16 µM/mg prot) (*p* < 0.05) (Figure 3C). 

Upon exposure of the airways to irritants such as ammonia, C-fibers are activated, leading to the release of SP and subsequent cough induction. In the model group, the SP level in BALF reached 11.69 pg/mL. This level was significantly reduced to 5.25 pg/mL in the positive control group and to 2.17 pg/mL in the AAPs-L group (*p* < 0.05). And no statistically significant difference in SP content was observed between the AAPs-H group and the model group (*p* > 0.05) (Figure 3D). The release of SP directly induces CXCL1 production, which recruits and activates neutrophils, leading to a substantial release of ROS and additional pro-inflammatory cytokines. This cascade establishes a positive feedback loop that exacerbates coughing. As shown in Figure 3E, the CXCL1 content in BALF was significantly lower in the AAPs-L group (439.11 pg/mL) compared with the model group (829.03 pg/mL) and the AAPs-H group (648.29 pg/mL) (*p* < 0.05), with no statistically significant difference observed relative to the positive control (658.41 pg/mL). Furthermore, as illustrated in Figure 3F, the level of the pro-inflammatory cytokine IL-1β was markedly reduced under AAPs-L treatment (214.25 pg/mL, model group 543.97 pg/mL) (*p* < 0.05). These results indicated that AAPs-L (200 mg/kg BW) alleviated the cough reflex in mice through mechanisms involving antioxidant activity and attenuation of inflammation.

### 3.4. Effects of AAPs on Expectorant Activity 

**Figure 4:**
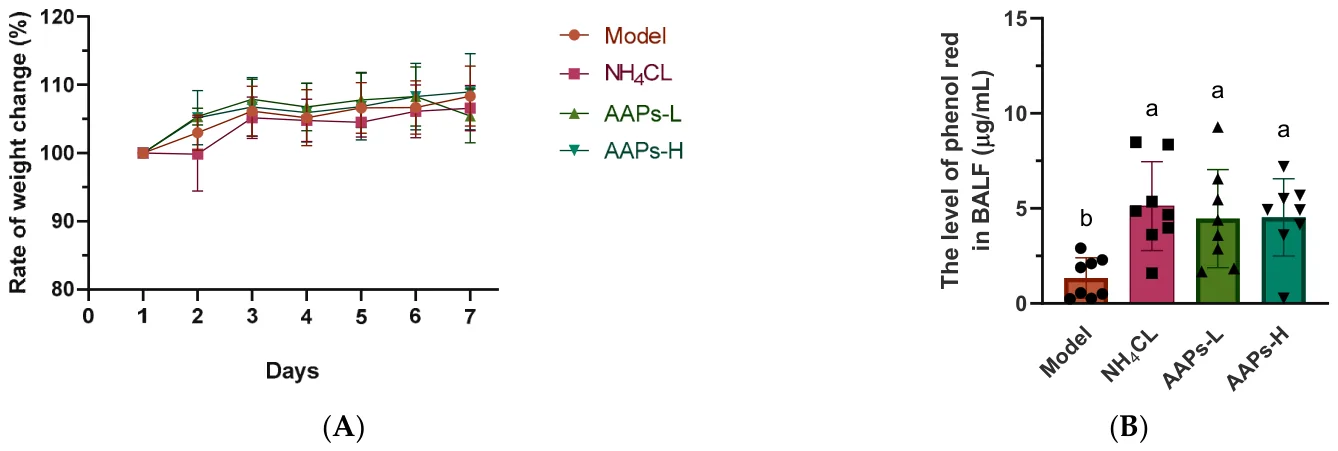
Effects of different molecular weight AAPs on rate of body weight change (**A**) and tracheal phenol red output in mice (**B**). Different lower-case letters correspond to significant differences at *p* < 0.05, *n* = 8.

The results of the expectorant test were shown in Figure 4. BW remained relatively stable across all groups during the expectorant test, with no statistically significant differences observed, indicating that the administration of AAPs-L and AAPs-H did not cause systemic injury or significant changes in BW (Figure 4A). The larger phenol red output indicated the stronger expectorant effect. As demonstrated in Figure 4B, compared with the model group, treatment with ammonium chloride, AAPs-L, and AAPs-H significantly enhanced tracheal phenol red output (*p* < 0.05), increasing the output by 2.79-fold, 2.31-fold, and 2.35-fold, respectively. These results indicated that 200 mg/kg BW of AAPs-L and AAPs-H demonstrated a similarly expectorant effects, promoting the production and clearance of respiratory tract mucus while exerting protective effects on the respiratory tract.

### 3.5. Effects of AAPs on Anti-Asthmatic Activity 

**Figure 5:**
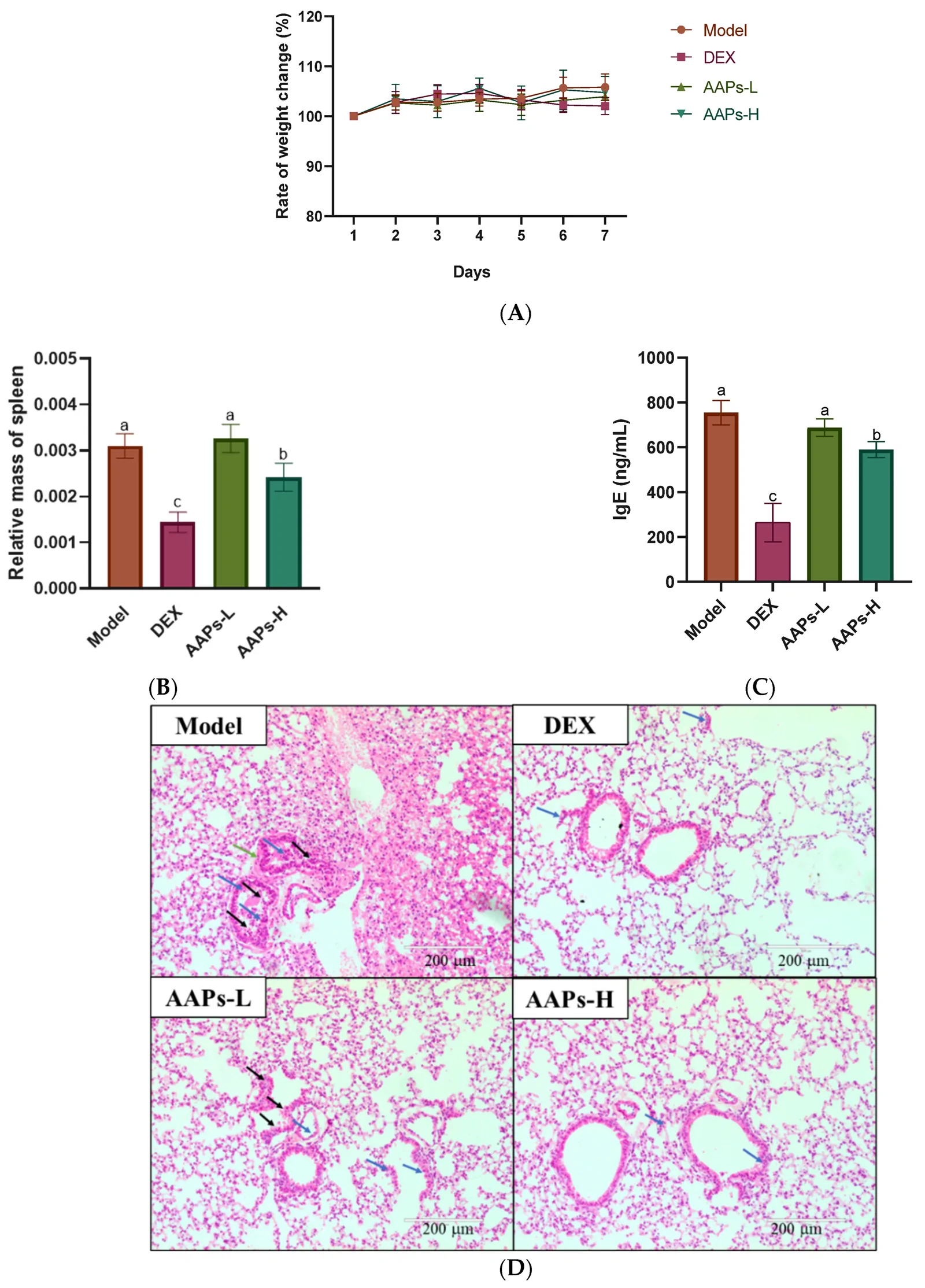

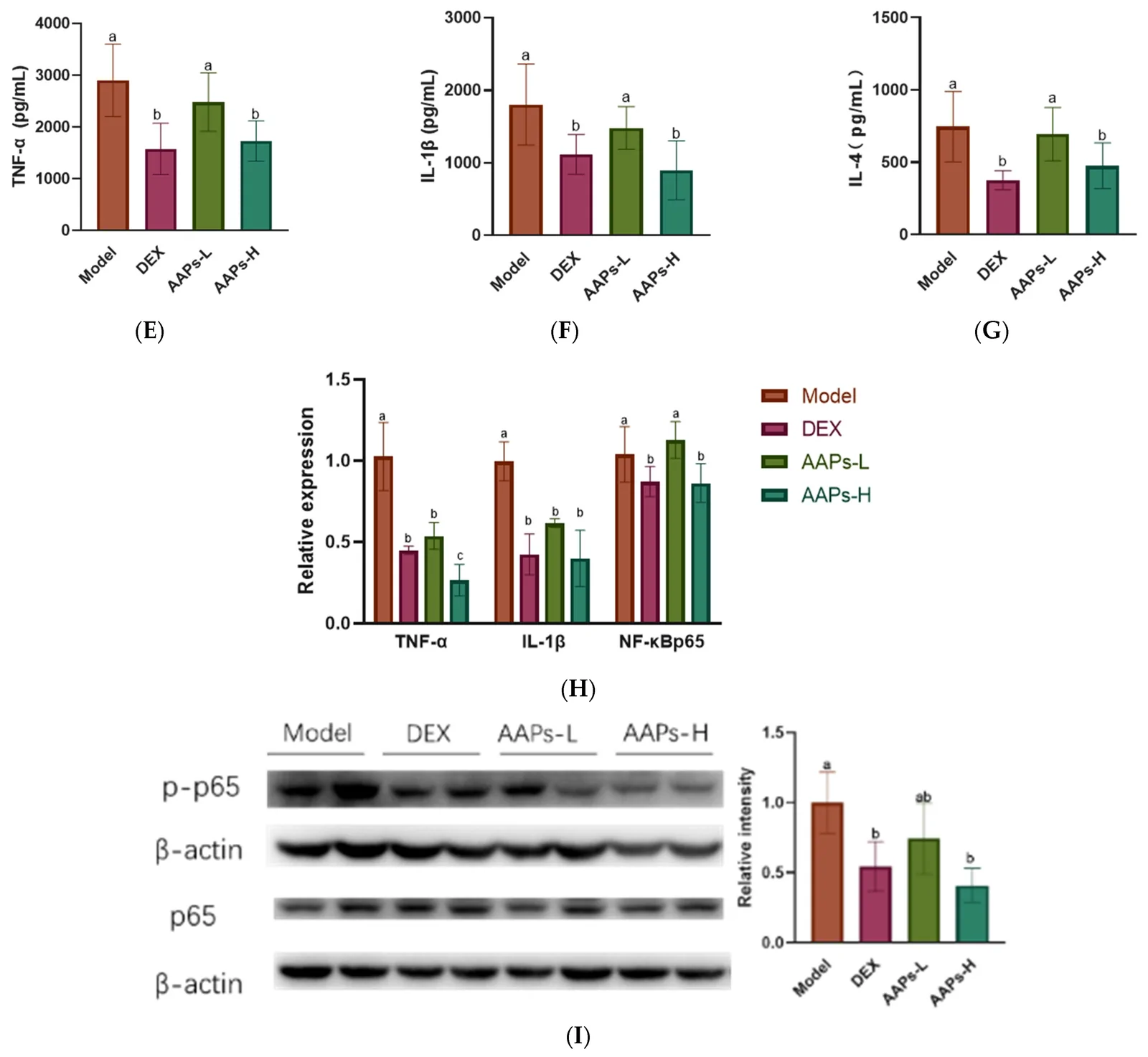
Effect of different molecular weight AAPs on anti-asthma activity in mice. (**A**) Rate of body weight change. (**B**) Relative mass of spleen. (**C**) IgE level in serum. (**D**) The H&E staining (magnification 200×) of lung tissue. The model control asthmatic group which was challenged with ovalbumin; The asthmatic group that received DEX; The tissue section from asthmatic mice treated with AAPs-L and AAPs-H. Black, blue, and green arrows represented of airway epithelial damage, inflammatory cell infiltration, and increased airway wall thickness, respectively. *n* = 3. (**E**) TNF-α, (**F**) IL-1β, and (**G**) IL-4 levels in BALF. (**H**) Relative expression of related genes in lung tissue based on qPCR. (**I**) Western blot analysis and quantification of NF-κB signaling pathway key proteins. Different lower-case letters correspond to significant differences at *p* < 0.05, *n* = 5, except for (**D**).

Anti-asthmatic activity of AAPs with different *M*_W_s was performed as described before. The results were shown in Figure 5. The BW of mice in all groups remained relatively stable throughout the test with no significant differences observed (Figure 5A). Additionally, before treatment, the latency to asthma onset in each group was approximately 36 s. Both AAPs-L and AAPs-H significantly prolonged the asthma induction latency, increasing it from 36.42 s to 46.74 s and from 36.12 s to 57.82 s, respectively (*p* < 0.05). No statistically significant difference was noted between these two treated groups (Table 2). AAPs-L and AAPs-H exhibited a notable inhibitory effect on asthma induced by OVA. Relative mass of spleen and serum IgE levels serve as primary indicators of systemic immune activation and inflammatory status in the OVA-induced asthma model. As shown in Figure 5B, the spleen index in the asthma model group was 2.2-fold higher than that in the DEX group, while the AAPs-H group showed a 22.6% reduction compared to the model group (*p* < 0.05). Correspondingly, serum IgE levels were significantly lower in the AAPs-H group (589.5 ng/mL) than in the model group (754.6 ng/mL) (*p* < 0.05, Figure 5C). However, the AAPs-L group exhibited no significant difference from the model group (*p* > 0.05), which contrasted with the results in the antitussive and expectorant experiments. AAPs-H (200 mg/kg BW) appeared to exert a stronger immunomodulatory potential against asthma in mice.

### 3.6. Analysis of AAPs on Histopathological Changes in Lung Tissue of Asthmatic Mice

H&E staining of lung tissues revealed that the asthma model group exhibited irregular bronchial epithelial arrangement, airway epithelial damage (black arrows), and substantial inflammatory cell infiltration (blue arrows), accompanied by a slight increase in airway wall thickness (green arrows). Both DEX and AAPs-H treatments markedly ameliorated these pathological alterations, with only minimal inflammatory cell infiltration observed (blue arrows). Furthermore, AAPs-L also notably attenuated the aforementioned pathological features, presenting only partial airway epithelial injury (black arrows) and inflammatory cell infiltration (blue arrows) (Figure 5D). As evidenced, both AAPs-L and AAPs-H could mitigate asthma-induced lung tissue damage to varying degrees, with AAPs-H demonstrating a more pronounced protective effect on the pulmonary histoarchitecture in asthmatic mice.

### 3.7. Effects of AAPs on Inflammatory Cytokine Expression and NF-κB Pathway in Asthmatic Mice

OVA-induced asthma is associated with alterations in inflammatory cytokines in BALF. In this study, levels of TNF-α, IL-1β, and IL-4 in BALF were measured across groups. As shown in Figure 5E–G, the Model group exhibited the highest concentrations of TNF-α (2901.67 pg/mL), IL-1β (1801.99 pg/mL), and IL-4 (746.65 pg/mL) among all groups (*p* < 0.05). DEX, as a glucocorticoid, significantly suppressed the expression of these inflammatory cytokines via transcriptional regulation (*p* < 0.05). Notably, the levels of TNF-α, IL-1β, and IL-4 in the AAPs-H group decreased to 1732.06 pg/mL, 898.81 pg/mL, and 475.88 pg/mL, respectively, showing no significant difference compared with the DEX group (*p* < 0.05). However, AAPs-L administration showed no significant difference compared to model mice (*p* > 0.05), a trend also observed in latency to asthma onset, spleen index, and serum IgE levels. Therefore, compared to AAPs-L, AAPs-H exerted a more potent anti-inflammatory effect by suppressing the release of the pro-inflammatory cytokines TNF-α and IL-1β, as well as the Th2-type cytokine IL-4, thereby effectively alleviating airway inflammation in asthmatic mice.

Further qPCR analysis of lung tissue elucidated the differential effects of AAPs with varying *M*_W_s on NF-κB pathway-associated gene expression in asthmatic mice (Figure 5H). Compared to the model group, both DEX and AAPs administration, regardless of *M*_W_, demonstrated the capacity to suppress pro-inflammatory cytokine expression to varying degrees. Notably, TNF-α, which is directly implicated in the development of airway hyperresponsiveness, exhibited a remarkable 74.8% reduction in mRNA expression in the AAPs-H group relative to the model group. This inhibitory effect not only surpassed that of the AAPs-L group (47.6% reduction) but also exceeded the efficacy of the positive control DEX group (56.3% reduction) (*p* < 0.05). Furthermore, mRNA levels of IL-1β were significantly downregulated by AAPs-H, AAPs-L, and DEX treatments (*p* < 0.05), reaching 39.85%, 61.6%, and 42.3% of the model group levels, respectively. The effect of AAPs on IL-1β expression was not significantly different from that of DEX (*p* > 0.05). The mRNA expression of NF-κB p65, a key upstream signaling molecule, was also reduced to 86.2% of the model level by AAPs-H, showing no significant difference from DEX treatment (87.1%) (*p* > 0.05). To investigate the mechanisms by which AAPs modulate NF-κB pathway activation and its pleiotropic transcriptional regulation in exerting anti-inflammatory effects, we analyzed the protein expression of p-p65 and NF-κB p65 in lung tissues via Western blot (Figure 5I). The p-p65/p65 ratio was significantly decreased to 54.4% of the model level in the DEX group (*p* < 0.05). The AAPs-H group showed a comparable effect to the positive control, reducing the ratio to 41% of the model level, while the AAPs-L group decreased it to 74.3%, which was not statistically significant versus the model group (*p* > 0.05). AAPs-H exhibited a greater tendency to reduce p-p65 expression compared with AAPs-L, although no significant difference was observed between these two groups (*p* > 0.05). Accordingly, AAPs-H (200 mg/kg BW) attenuated airway inflammation in asthmatic mice, primarily by inhibiting the NF-κB pathway activation, thereby suppressing the transcription and expression of downstream pro-inflammatory cytokines, including TNF-α and IL-1β.

### 3.8. Effects of AAPs on the Gut Microbiota of Asthmatic Mice

**Figure 6:**
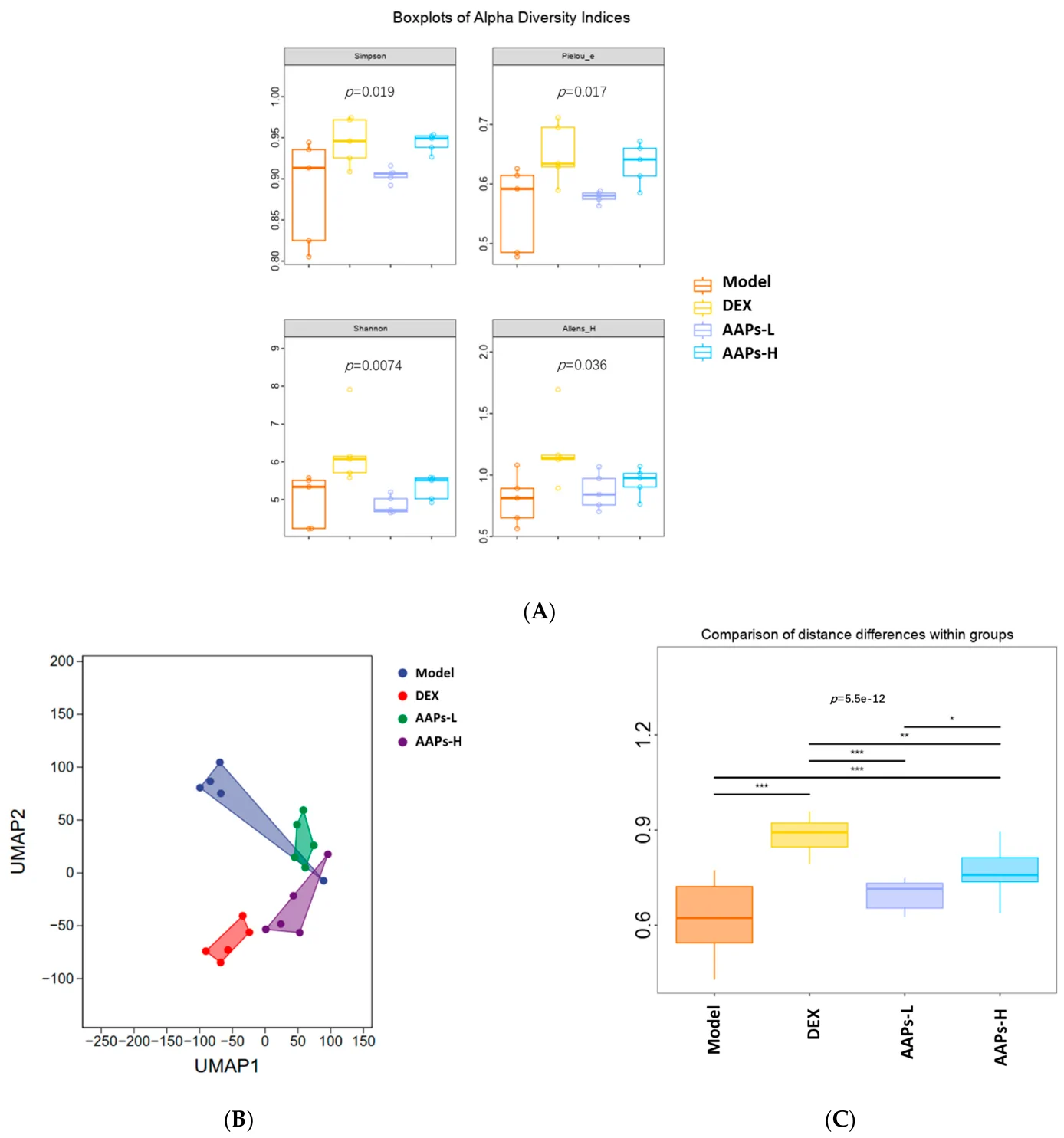

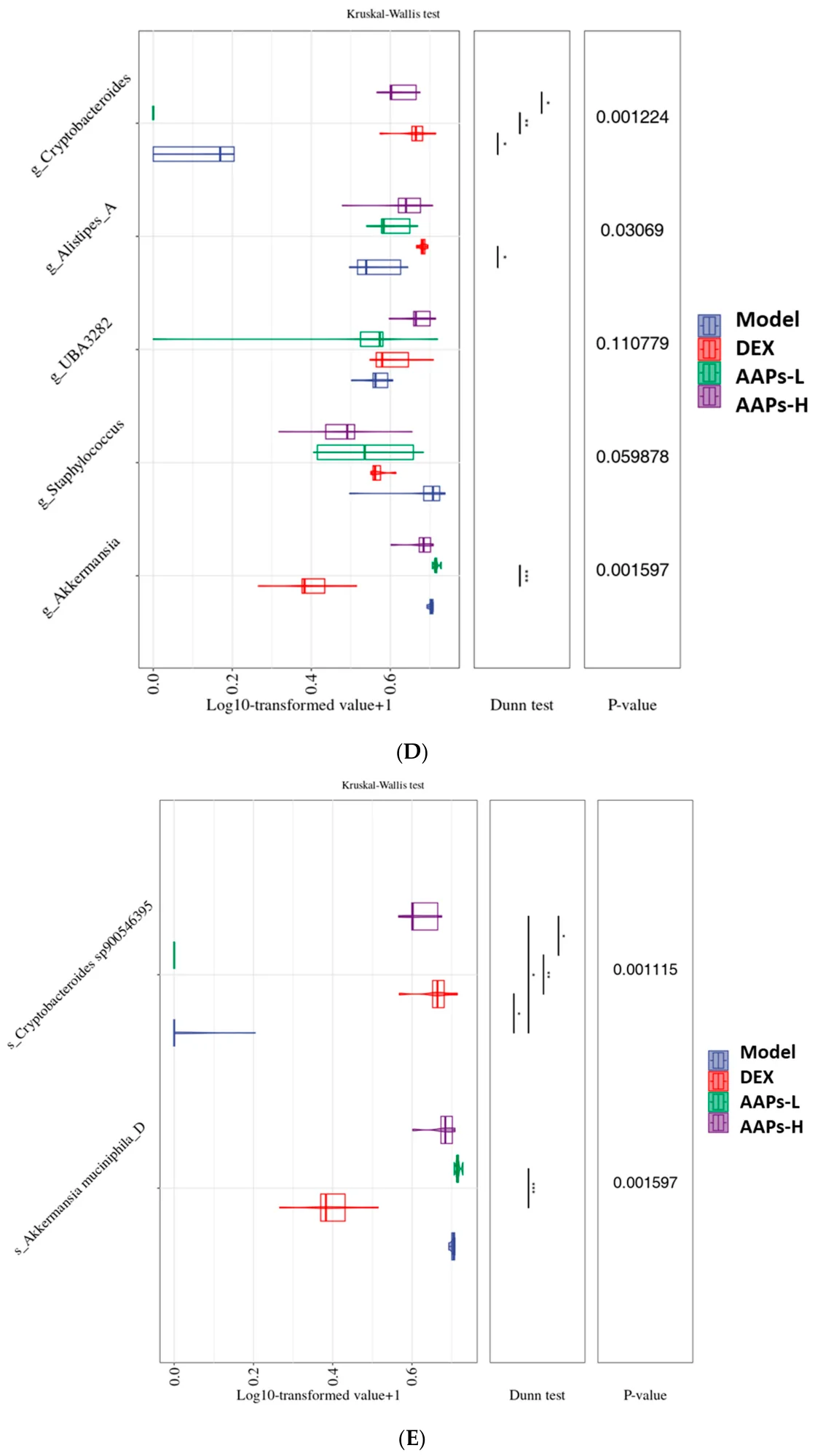

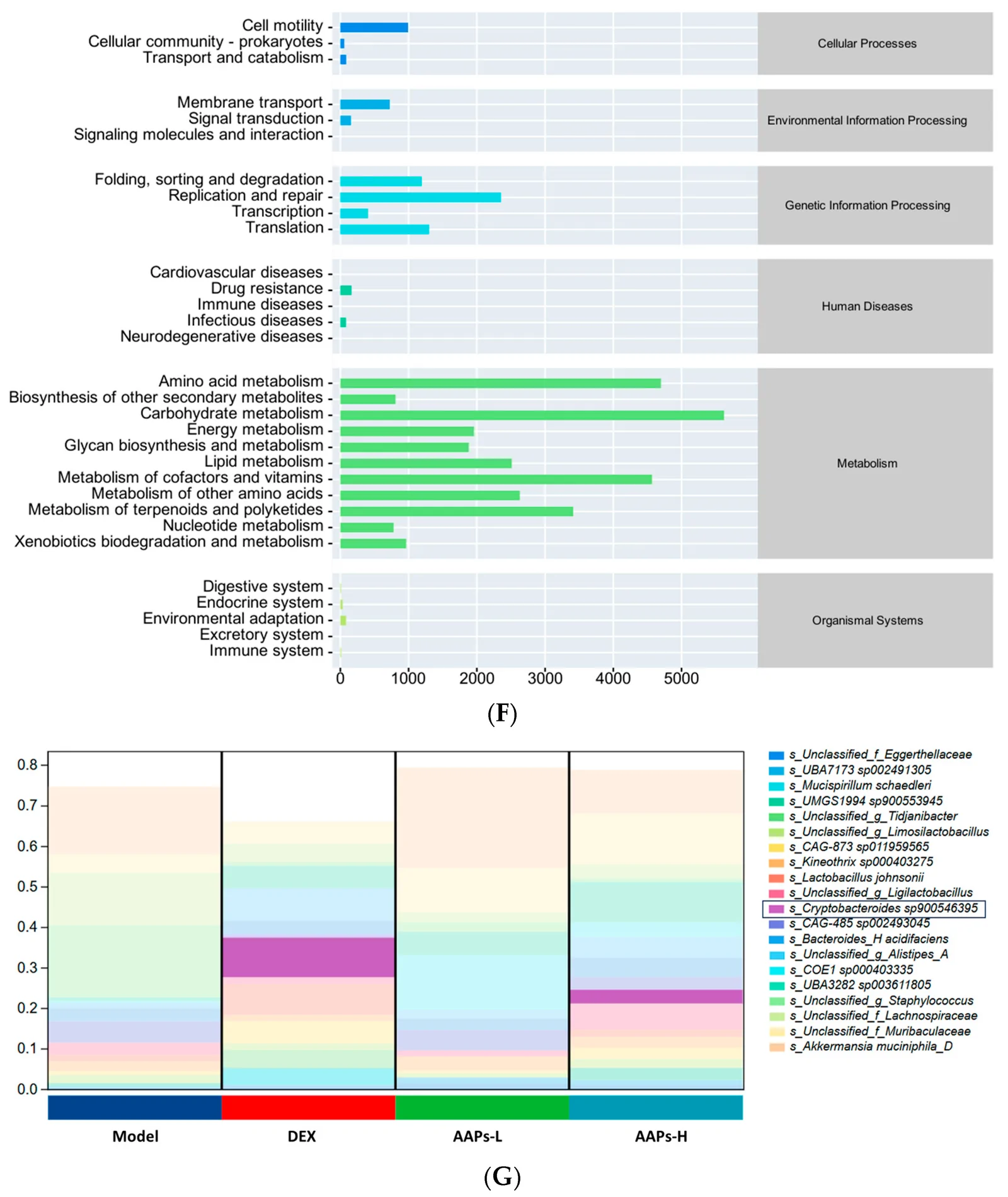
Effects of different molecular weight AAPs on the composition and functional pathways of gut microbiota in asthmatic mice. (**A**) Boxplots of Alpha diversity indices, (**B**) tSNE scatter plot of the gut microbiota, (**C**) intergroup difference analysis, (**D**) relative abundance of gut microbial at the genus level between groups, including *Cryptobacteroides, Alistipes_A, UBA3282, Staphylococcus* and *Akkermansia*, (**E**) relative abundance of gut microbial at the species level between groups, including *Cryptobacteroides sp900546395* and *Akkermansia muciniphila_D*, (**F**) metabolic pathway (KEGG database) statistics, and (**G**) species composition analysis based on kegg metabolic. * *p* < 0.05, ** *p* < 0.01, *** *p* < 0.001 indicated significant differences in pairwise comparisons. Further, we visualized the compositional distribution of intestinal content samples across four groups at the genus level to determine which ASV account for differences in composition of the gut microbial community. Analysis of the relative abundance bar plots (top 20 genera) revealed variations in the abundances of *Akkermansia*, *Staphylococcus*, *UBA3282*, *Alistipes_A*, and *Cryptobacteroides* (Figure S4B). Subsequent Kruskal–Wallis tests indicated that *Cryptobacteroides*, *Alistipes_A*, and *Akkermansia* exhibited significant intergroup abundance differences. Notably, both AAPs-H and DEX treatments significantly increased the abundance of *Cryptobacteroides* in the gut microbiota of asthmatic mice (**D**). To further delineate the specific regulatory effects of AAPs-H on gut microbiota at the species level, the top 20 species were plotted (Figure S4C). This analysis showed that AAPs-H and DEX significantly elevated the abundance of *Cryptobacteroides sp900546395* (*p* = 0.001115), while the abundance of *Akkermansia muciniphila_D* was significantly reduced in the DEX group (*p* = 0.001597) (**E**).

The effect of AAPs with different *M*_W_s on the gut microbiota of asthmatic mice was evaluated by sequencing the V3-V4 variable regions of the bacterial 16S rRNA gene in intestinal contents. As shown by the species accumulation curves in the α-diversity analysis, the curves for the gut microbial samples from the four groups gradually plateaued with increasing sample size (Figure S4A), indicating that the sample size used in this study (*n* = 5) was sufficient to reflect the diversity of gut microbiota across different treatment groups, thereby ensuring the reliability of subsequent analyses. Figure 6A presented differences in α-diversity indices. Compared with the model group, both AAPs-H and AAPs-L groups, similar to the DEX group, exhibited significant increases in microbial diversity (Simpson and Shannon indices), evenness (Pielou’s evenness index), and phylogeny-based diversity (Allen’s H), indicating that AAPs could significantly alleviate asthma-induced reduction in gut microbial diversity in mice. Similarly, in β-diversity analysis, dimensionality reduction revealed distinct clustering of gut microbiota among the different groups in the tSNE clustering map (Figure 6B), demonstrating clear structural differences in the gut microbial composition across the four groups. The AAPs-H group showed greater spatial separation from the model group than the AAPs-L group, indicating more pronounced differences and a degree of uniqueness. Furthermore, box-plot analysis of inter-group distances based on β-diversity demonstrated a highly significant difference (*p* < 0.001) between the AAPs-H group and the asthma model group (Figure 6C). 

Finally, PICRUSt analysis was employed to predict the functional potential of the gut microbiota. Based on the KEGG database, the abundance of metabolic pathways annotated by microbial gene functions is presented in Figure 6F. At Level 1, the pathways for “Metabolism” and “Genetic Information Processing” were significantly altered, primarily involving “Carbohydrate metabolism,” “Amino acid metabolism,” “Metabolism of cofactors and vitamins,” and “Replication and repair.” Notably, species contribution analysis for the “Metabolism” pathway revealed that *Cryptobacteroides sp900546395* contributed a higher proportion in asthmatic mice treated with DEX or AAPs-H compared to the model and AAPs-L groups (Figure 6G). These findings suggested that 200 mg/kg BW AAPs-H might alleviate asthma via reshaping the gut microbiota composition, specifically by increasing the abundance of *Cryptobacteroides sp900546395*, thereby influencing related functional pathways such as “Metabolism” and “Genetic Information Processing.”

## 4. Discussion

*A. auricula* is a medicinal and edible fungus with a history of use in China spanning thousands of years. Its primary bioactive components are AAPs, whose antioxidant, anti-inflammatory, and immunomodulatory activities have been extensively documented [34,35]. Notably, AAPs with different *M*_W_s exhibit varying tendencies in biological activity. For instance, He et al. compared the free radical-scavenging capacity and nitric oxide production in macrophages of AAPs with varying *M*_W_s obtained through different extraction methods [36]. The present study reveals, for the first time, the differential protective effects of AAPs with distinct *M*_W_s against respiratory disorders. Specifically, AAPs-L (31 kDa) demonstrated superior antitussive activity, attributed to its stronger direct antioxidant and anti-inflammatory capacities. In contrast, AAPs-H (1500 kDa) might be more effective at alleviating airway inflammation in asthma through immunomodulation and remodeling of gut microbiota.

AAPs are composed of a series of polysaccharide molecules with varying *M*_W_s. Different extraction methods can cause varying degrees of damage to the cell wall, resulting in different compositions of polysaccharides, and the molecular chains of polysaccharides may also undergo cleavage during the extraction process. In this study, H_2_O_2_ treatment was employed to cleave glycosidic bonds via hydroxyl (-OH) radicals, reducing the *M*_W_ of AAPs from 1554 kDa to 31 kDa. It should be noted that at least a portion of the AAPs-L may be generated via chemical degradation and structural modification during processing. This treatment enhanced solubility, reduced viscosity, and facilitated rapid penetration into the reaction system, allowing exposure to free radicals (ABTS^+^•, DPPH•, O_2_^−^•) and promoting the availability of reducing ends and active groups such as -OH moieties. Through electron transfer or hydrogen atom donation, these active groups neutralize free radicals, thereby terminating the radical chain reaction [37]. Consequently, AAPs-L exhibited superior in vitro antioxidant activity compared to AAPs-H, consistent with findings reported by Ma et al. [38], who demonstrated that lower-*M*_W_ degraded AAPs display significantly enhanced free radical-scavenging capacity. It is important to note that the influence of *M*_W_ on the bioactivity of AAPs should be considered in conjunction with other structural factors, among which monosaccharide composition plays a critical role in the structure-activity relationship. In the present study, the Glc content of AAPs-L was nearly twice that of AAPs-H. Studies have indicated that the abundance of Glc may be attributed to the most bioactivities of AAPs [10], as a pyranose monosaccharide, Glc possesses multiple -OH groups that serve as typical antioxidant active sites. Moreover, the Man-to-Glc ratio in AAPs-L was approximately 1:1.28, which is more balanced than that in AAPs-H (approximately 1:0.5). It has been reported that a balanced Man-to-Glc ratio may optimize the spatial conformation of polysaccharides [39], potentially reducing steric hindrance at active sites and further enhancing free radical binding efficiency. Unlike some findings, AAPs-H contained a higher proportion of Glc-UA but did not exhibit superior antioxidant activity [40]. This discrepancy may be explained by the possibility that *M*_W_ and the spatial distribution of Glc-UA are more critical determinants than its total content. Supporting this view, Zhao et al. reported that a low-*M*_W_ fraction of *Laminaria japonica* sulfated polysaccharides (LM1, Mp 1.5 kDa) retained notable free radical scavenging capacity despite containing negligible amounts of Glc-UA [41]. Meanwhile, the higher Man content in AAPs-H suggested favorable immunomodulatory potential, as immune cells can recognize AAPs through Man receptors, thereby stimulating cytokine production. In contrast, the lower Man content in AAPs-L corresponded with higher solubility and lower viscosity, consistent with observations by Hoseinpour et al. [39]. Accordingly, the differences in activity among these AAPs with different *M*_w_s cannot be attributed exclusively to *M*_w_. Collectively, these physicochemical properties highlight the AAPs’ promising potential for diverse future applications.

This superior in vitro antioxidant activity provides a material basis for the direct antitussive and expectorant effects of AAPs in vivo, particularly for AAPs-L. The antitussive effect of plant polysaccharides based on their antioxidant properties has been well established [42,43]. ROS and their downstream lipid peroxidation products (e.g., MDA) can directly activate the transient receptor potential A1 (TRPA1) channel, leading to depolarization of sensory neurons (C-fibers) and subsequent release of neuropeptides such as SP, thereby triggering cough. SP, in turn, induces a cascade of pro-inflammatory mediators, including cytokines and chemokines such as IL-1 and CXCL1, which further promote ROS release. This positive feedback loop results in sustained elevation of cough sensitivity [44,45]. The potent antioxidant activity of AAPs-L can directly scavenge excessive ROS in the airways, enhance antioxidant enzyme activity, and restore redox homeostasis, thereby reducing SP release and CXCL1 expression and subsequently alleviating oxidative stress. Thus, AAPs-L may alleviate cough via an antioxidant-dependent mechanism involving the ROS-SP-CXCL1 axis. However, direct evidence for this causal pathway is lacking and requires further verification. Moreover, owing to its low *M*_W_, AAPs-L may rapidly penetrate the airway mucus layer and adhere to the surface of airway epithelial cells [46], forming a protective barrier that reduces direct mucosal irritation by ammonia [47]. A similar mechanism may support the expectorant effect of AAPs [48].

Unlike its antitussive effect, AAPs-H exhibited a more apparent anti-inflammatory trend in the more complex asthma model, which is characterized by chronic airway inflammation involving not only inflammation but also immune imbalance and systemic dysregulation [49]. Cytokines, inflammatory cells and inflammatory mediators interact with each other, resulting in the infiltration of a large number of airway eosinophils and CD4+ T cells, as well as mucus hypersecretion, airway hyperresponsiveness, airway remodeling and increased IgE production. An imbalance in the cytokine levels produced by Th1 and Th2 cells leads to asthma [50]. Th2-type cytokines have been implicated in promoting allergic responses in asthma, such as IL-4 [51], which may induce the production and aggregation of eosinophils, as well as stimulate B cells to produce IgE. AAPs-H restored the balance of Th1 and Th2 immunity by reducing the levels of IL-4 and TNF-α. Consequently, the serum IgE levels were also reduced after AAPs-H treatment. These immunomodulatory effects of polysaccharides have been documented [51,52]. And high-*M*_W_ polysaccharides are more likely to induce receptor cross-linking due to their structural complexity, and immune cells can recognize AAPs via Man receptors to stimulate cytokine production [10]. Therefore, the superior immunomodulatory activity of AAPs-H compared to AAPs-L may be attributed to its higher *M*_W_ and greater Man content. 

In addition, AAPs-H reduced NF-κB phosphorylation, although no statistically significant difference in p-p65 expression was detected between AAPs-H and AAPs-L, AAPs-H showed a stronger trend toward suppressing NF-κB activation. Further, AAPs-H suppressed the expression of pro-inflammatory cytokines, including TNF-α and IL-1β, consistent with the anti-inflammatory mechanism of AAPs reported by Xiang et al. [53]. This modulation of the NF-κB signaling pathway may occur indirectly via the gut microbiota-immune axis [54]. The high *M*_W_ of AAPs-H allows it to reach the colon more efficiently, where it acts as a prebiotic to selectively promote the growth of specific beneficial bacteria. In our work, the concurrent increase in *Cryptobacteroides sp900546395* abundance in both AAPs-H and DEX groups supported its association with beneficial outcomes. Although the precise immunomodulatory mechanisms of *Cryptobacteroides sp900546395* remain to be elucidated, the abundance of the *Cryptobacteroides* genus is negatively correlated with inflammation [55], and its members are capable of producing short-chain fatty acids (SCFAs), which are well-established mediators of anti-inflammatory effects in the gut-lung axis [56]. Our future studies will aim to demonstrate the potential of *Cryptobacteroides* sp. *900546395* to mediate asthma remission via the gut-lung axis.

## 5. Conclusions

In summary, this study prepared and characterized two AAPs with distinct *M*_W_s and compositions, and evaluated their protective effects on the respiratory system. The results demonstrated that AAPs-L (31 kDa) and AAPs-H (1554 kDa) both alleviated cough in mice, and the stronger antioxidant capacity of AAPs-L may contribute to this effect, making it significantly more effective than AAPs-H in prolonging the cough latency. In the anti-asthmatic model, AAPs-H tended to show greater benefits by alleviating airway inflammation in asthmatic mice and exhibiting an anti-asthmatic tendency, which were associated with restoring Th1/Th2 immune balance, modulating the NF-κB signaling pathway, and reshaping the gut microbiota. These findings provide a new strategy for functional food development and comprehensive utilization of AAPs with different *M*_W_s.

## Figures and Tables

**Table 1:** Monosaccharide composition (molar ratios).

Sample	Fuc	Rha	Ara	Gal	Glc	Xyl	Man	Glc-UA
AAPs-H	1.57%	0.15%	0.17%	2.17%	24.21%	10.15%	50.24%	11.33%
AAPs-L	0.98%	0.12%	0.14%	1.29%	46.01%	7.77%	36.33%	7.35%

Note: Fuc, fucose; Rha, rhamnose; Ara, arabinose; Gal, galactose; Glc, glucose; Xyl, xylose; Man, mannose; Glc-UA, glucuronic acid. Values are expressed as relative molar percentages.

**Table 2:** Latency to asthma onset in mice of different groups (s).

	Model	DEX	AAPs-L	AAPs-H
Before treatment	36.22 ± 2.52 ^c^	35.90 ± 1.74 ^c^	36.42 ± 1.88 ^c^	36.12 ± 1.69 ^c^
After treatment	42.6 ± 2.35 ^c^	81.23 ± 4.12 ^a^	46.74 ± 6.98 ^bc^	59.82 ± 3.98 ^b^

Different lower-case letters correspond to significant differences at *p* < 0.05, *n* = 5.

## Data Availability

The original contributions presented in this study are included in the article/supplementary material. Further inquiries can be directed to the corresponding authors.

## Supplementary Materials

The following supporting information can be downloaded at: https://www.mdpi.com/article/10.3390/foods15213833/s1, Figure S1: Yields of AAPs-H and AAPs-L; Figure S2: Ion chromatograms of monosaccharide standards (A), AAPs-H (B), and AAPs-L (C); Figure S3: Effect of different molecular weight AAPs on the ammonia liquor induced cough in mice. (A) The relative mass of liver and (B) thymus. Different lower-case letters correspond to significant differences at *p* < 0.05, *n* = 8; Figure S4: Effects of different molecular weight AAPs on the composition and functional pathways of gut microbiota in asthmatic mice. (A) Species accumulation curve, (B) the top 20 species composition differences between different groups at the genus level, and (C) the top 20 species composition differences between different groups at the species level. *n* = 5; Table S1: Concentrations, retention times and peak areas of standard monosaccharides.
