# Supplementary material for: Comparative Respiratory Protective Effects of Auricularia auricula Polysaccharide Fractions: From Antioxidant-Dependent Antitussive Effects to Microbiota-Associated Anti-Asthmatic Activity
Source: Foods. 2026 Oct 27;15(21):3833. doi: 10.3390/foods15213833 (PMC13762607; doi:10.3390/foods15213833)
Supplement: Supplementary file 1 [file foods-15-03833-s001.zip › foods-4503608-supplementary.pdf]

Supplementary

Figure captions

Figure S1. Yields of AAPs-H and AAPs-L

Figure S2. Ion chromatograms of monosaccharide standards (A), AAPs-H (B), and AAPs-L (C)

Figure S3. Effect of different molecular weight AAPs on the ammonia liquor induced cough in mice. (A) The relative mass of liver and (B) thymus. Different lower-case letters correspond to significant differences at $p < 0.05$, $n = 8$.

Figure S4. Effects of different molecular weight AAPs on the composition and functional pathways of gut microbiota in asthmatic mice. (A) Species accumulation curve, (B) the top 20 species composition differences between different groups at the genus level, and (C) the top 20 species composition differences between different groups at the species level. $n = 5$.

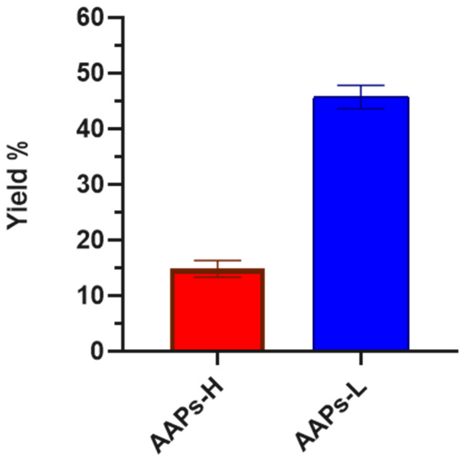
Yield %
0
10
20
30
40
50
60
AAPs-H
AAPs-L

Figure S1

A

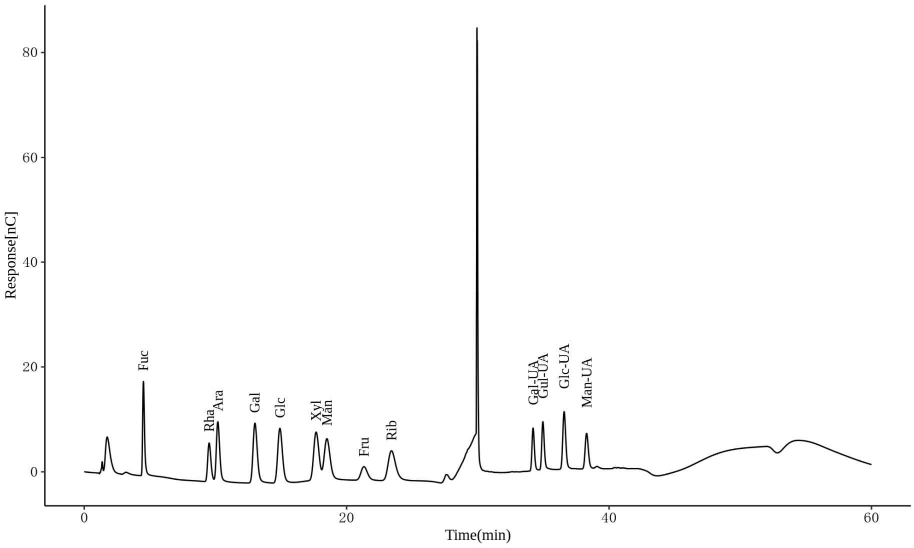
Response[nC]
Time(min)
Fuc
Rha
Ara
Gal
Glc
Xyl
Man
Fru
Rib
Gal-UA
Gul-UA
Glc-UA
Man-UA

B

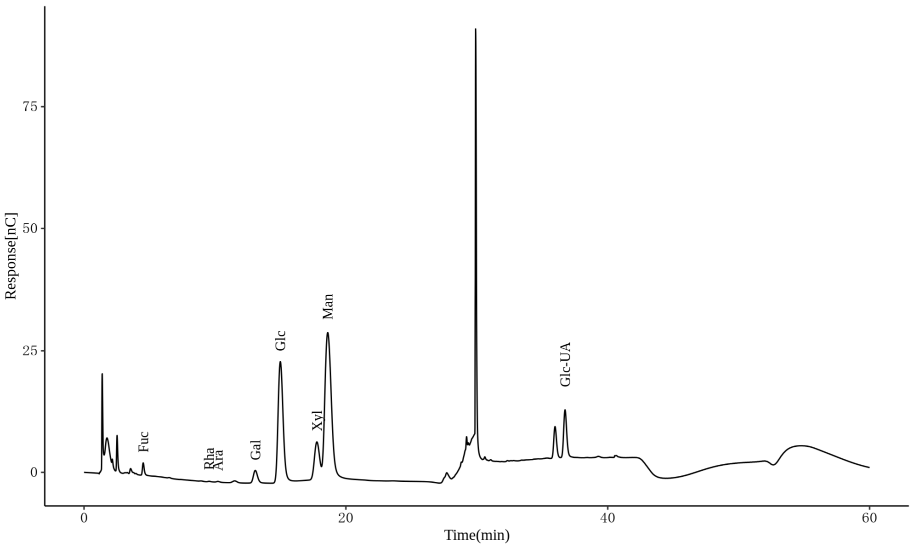
Response[nC]
Time(min)
Fuc
Rha
Ara
Gal
Glc
Xyl
Man
Glc-UA

C

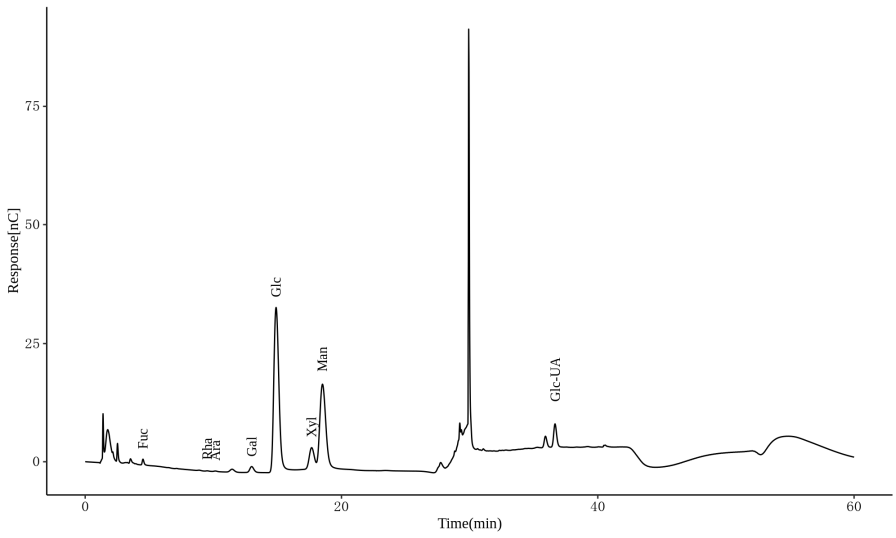


Figure S2

A

B

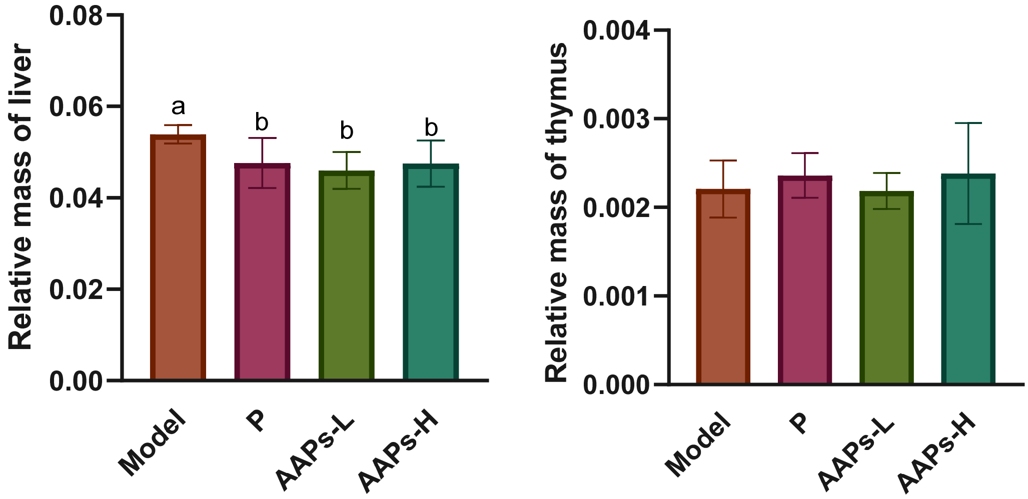


Figure S3

A

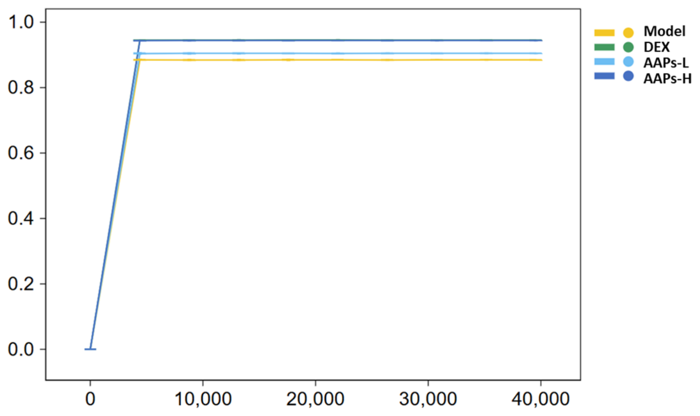

B

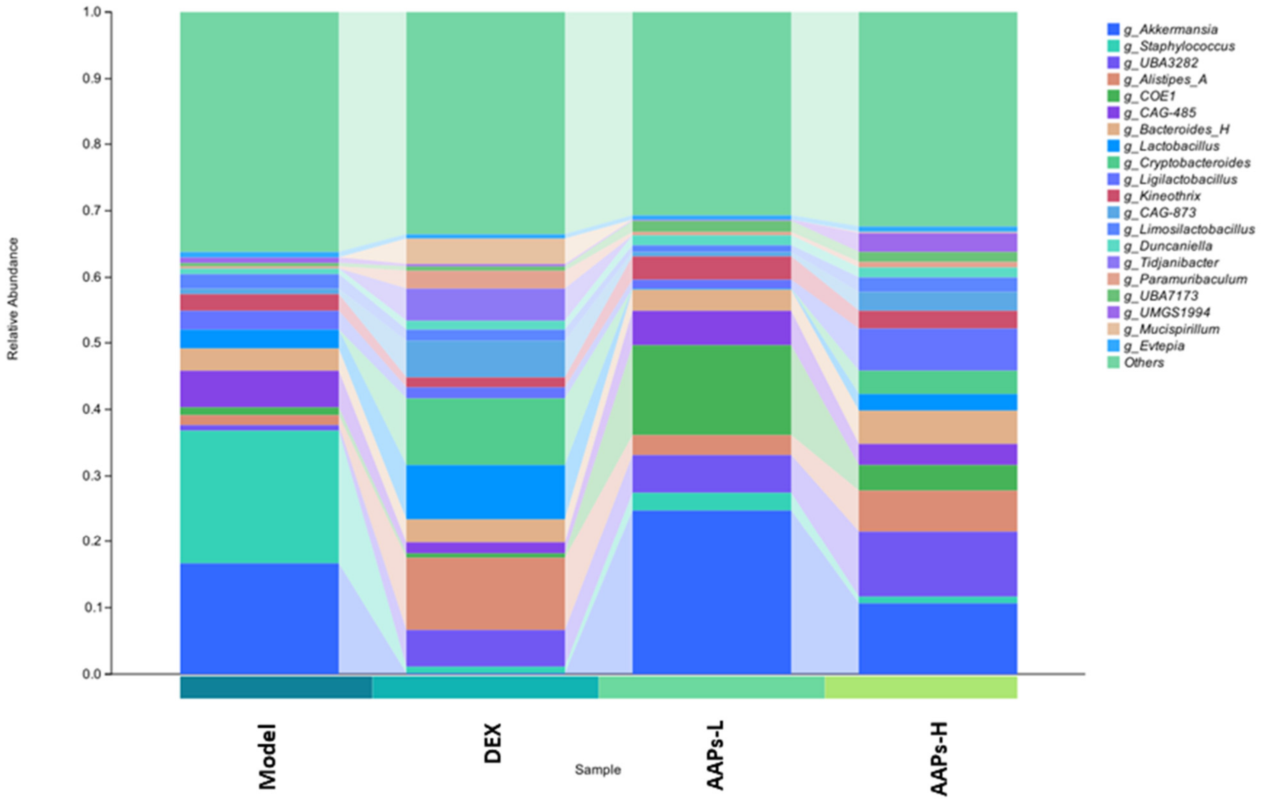

Relative Abundance
1.0
0.9
0.8
0.7
0.6
0.5
0.4
0.3
0.2
0.1
0.0
Model
DEX
Sample
AAPs-L
AAPs-H
g_Akkermansia
g_Staphylococcus
g_UBA3282
g_Alistipes_A
g_COE1
g_CAG-485
g_Bacteroides_H
g_Lactobacillus
g_Cryptobacteroides
g_Ligilactobacillus
g_Kineothrix
g_CAG-873
g_Limosilactobacillus
g_Duncaniella
g_Tidjanibacter
g_Paramuribaculum
g_UBA7173
g_UMGS1994
g_Mucispirillum
g_Evtepia
Others


C

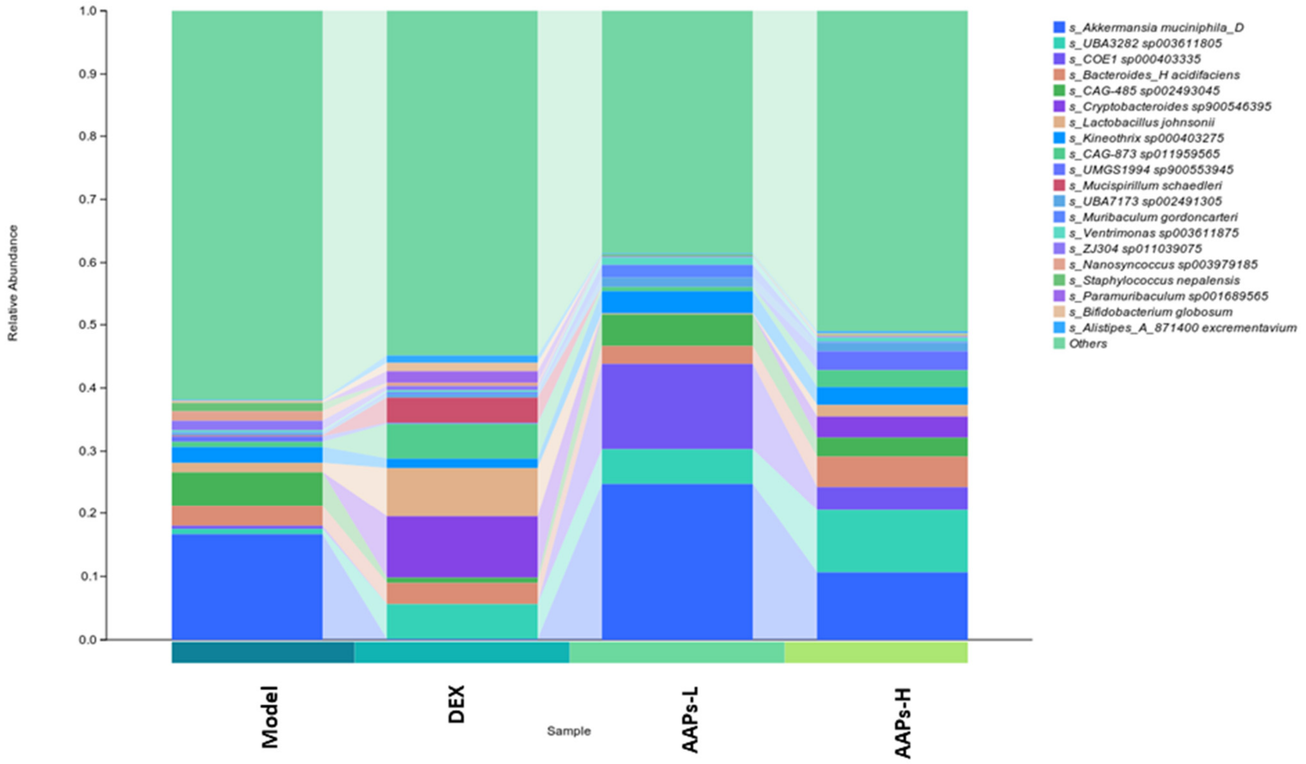

Relative Abundance
1.0
0.9
0.8
0.7
0.6
0.5
0.4
0.3
0.2
0.1
0.0
Model
DEX
Sample
AAPs-L
AAPs-H
s_Akkermansia muciniphila_D
s_UBA3282 sp003611805
s_COE1 sp000403335
s_Bacteroides_H acidifaciens
s_CAG-485 sp002493045
s_Cryptobacteroides sp900546395
s_Lactobacillus johnsonii
s_Kineothrix sp000403275
s_CAG-873 sp011959565
s_UMGS1994 sp900553945
s_Mucispirillum schaedleri
s_UBA7173 sp002491305
s_Muribaculum gordoncarteri
s_Ventrimonas sp003611875
s_ZJ304 sp011039075
s_Nanosyncoccus sp003979185
s_Staphylococcus nepalensis
s_Paramuribaculum sp001689565
s_Bifidobacterium globosum
s_Alistipes_A_871400 excrementavium
Others


Figure S4

Table S1 Concentrations, retention times and peak areas of standard monosaccharides

| **No.** | **Name** | **Name** | **concentration（mg/L）** | **RT（min）** | **Area** |
|---|---|---|---|---|---|
| **1** | Fucose | Fuc | 0.8 | 4.500 | 14.671 |
| **2** | Rhamnose | Rha | 0.8 | 9.492 | 10.258 |
| **3** | Arabinose | Ara | 0.8 | 10.150 | 18.054 |
| **4** | Galactose | Gal | 0.8 | 12.975 | 22.350 |
| **5** | Glucose | Glc | 0.8 | 14.875 | 23.228 |
| **6** | Xylose | Xyl | 0.8 | 17.617 | 21.475 |
| **7** | Mannose | Man | 1 | 18.425 | 23.038 |
| **8** | Fructose | Fru | 1.2 | 21.200 | 8.972 |
| **9** | Ribose | Rib | 1.2 | 23.284 | 21.238 |
| **10** | Galacturonic acid | Gal-UA | 1.2 | 34.167 | 10.304 |
| **11** | Guluronic acid | Gul-UA | 1.2 | 34.909 | 12.473 |
| **12** | Glucuronic acid | Glc-UA | 1.2 | 36.517 | 17.215 |
| **13** | Mannuronic acid | Man-UA | 1.2 | 38.225 | 11.075 |
